# Activation of RhoC by regulatory ubiquitination is mediated by LNX1 and suppressed by LIS1

**DOI:** 10.1038/s41598-022-19740-1

**Published:** 2022-10-03

**Authors:** Stanislav Kholmanskikh, Shawn Singh, M. Elizabeth Ross

**Affiliations:** Center for Neurogenetics, Feil Family Brain and Mind Research Institute, Weill Cornell Medicine, 413 East 69th St, Box 240, New York, NY 10021 USA

**Keywords:** Biochemistry, Cell biology, Neuroscience, Molecular medicine, Neurology, Oncology

## Abstract

Regulation of Rho GTPases remains a topic of active investigation as they are essential participants in cell biology and the pathophysiology of many human diseases. Non-degrading ubiquitination (NDU) is a critical regulator of the Ras superfamily, but its relevance to Rho proteins remains unknown. We show that RhoC, but not RhoA, is a target of NDU by E3 ubiquitin ligase, LNX1. Furthermore, LNX1 ubiquitination of RhoC is negatively regulated by LIS1 (aka, PAFAH1B1). Despite multiple reports of functional interaction between LIS1 and activity of Rho proteins, a robust mechanism linking the two has been lacking. Here, LIS1 inhibition of LNX1 effects on RhoGDI-RhoC interaction provides a molecular mechanism underpinning the enhanced activity of Rho proteins observed upon reduction in LIS1 protein levels. Since LNX1 and RhoC are only found in vertebrates, the LIS1-LNX1-RhoC module represents an evolutionarily acquired function of the highly conserved LIS1. While these nearly identical proteins have several distinct RhoA and RhoC downstream effectors, our data provide a rare example of Rho-isoform specific, upstream regulation that opens new therapeutic opportunities.

## Introduction

Significant advances in our understanding of Rho GTPase function and regulation have been made since their discovery in the early 1980s^1^. The involvement of Rho GTPases has been documented in numerous human diseases, including cancer, vascular disease, brain malformations, neurodevelopmental disorders in the autism spectrum, and neurodegenerative diseases^2–5^. Despite these insights, therapies targeting Rho proteins have been elusive at best^6^. This is in part because of the pervasive actions of Rho GTPases that hinder the ability to selectively target a particular pathogenic process. Thus, further characterization of the regulatory mechanisms controlling Rho proteins will both advance our knowledge of cell biology and may offer novel therapeutic opportunities. In this study, we asked whether Rho proteins might be targets of regulatory non-degrading ubiquitination (NDU) and/or SUMOylation, similar to other GTPases in the Ras superfamily^7,8^. We report here a previously unrecognized pathway to show RhoC, but not RhoA, is positively regulated by E3 ubiquitin ligase LNX1-dependent NDU, elucidate the mechanism of this activation by LNX1 and demonstrate its suppression by LIS1.

## Results

**Figure 1:**
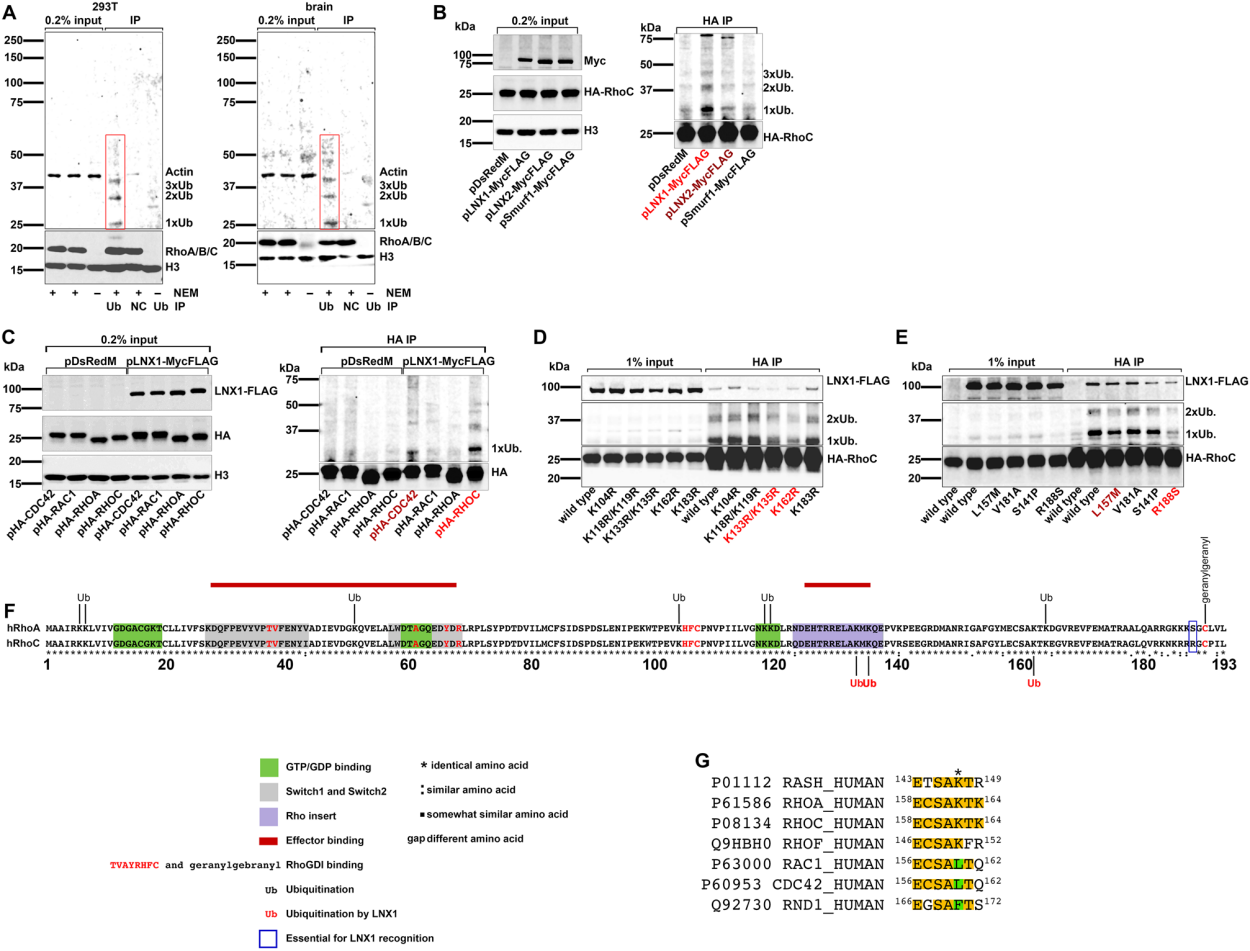
RhoC is targeted by LNX1 for monoubiquitination. (**A**) Ubiquitin binding domain pull down from HEK293T cells or whole mouse brain. Immunoblots using RhoA/B/C antibody reveals four bands. The strongest band runs at 21 kDa and corresponds to unmodified Rho protein. Three higher molecular weight bands run at 28, 35 and 42 kDa and correspond in size to mono-, di- and tri-ubiquitinated Rho protein respectively. No such higher MW bands are detected in the negative control (control beads without ubiquitin binding domain) or when N-ethylmaleimide (NEM), covalent inhibitor of cysteine proteases, is omitted from lysis buffer resulting in the rapid removal of ubiquitin by deubiquitinating enzymes. (**B**) HA co-immunoprecipitation from co-transfected HEK293T cells expressing 3xHA RhoC and FLAG epitope-tagged proteins as indicated (or pDsRedM as negative control). LNX1 and to a lesser degree LNX2 induce RhoC monoubiquitination. 3xHA-RhoC(Ub_1_) is present at low level in the control transfected cells or cells co-transfected with Smurf1. (**C**) HA co-immunoprecipitation from co-transfected HEK293T cells expressing LNX1-FLAG epitope tagged and 3HA tagged proteins as indicated (or pDsRedM as negative control). Right panel in C.: LNX1 mediates robust monoubiquitination of RhoC and to a lesser degree Cdc42, but not Rac1 or RhoA. (**D**) HA co-immunoprecipitation from co-transfected HEK293T cells expressing LNX1-FLAG epitope tagged and 3HA tagged WT or mutant RhoC proteins as indicated. Only mutation of K133/K135 or K162R reduces RhoC mono-ubiquitination. (**E**) HA co-immunoprecipitation from co-transfected HEK293T cells expressing LNX1-FLAG epitope tagged and 3HA tagged WT or mutant RhoC proteins as indicated. The only RhoC mutation causing nearly complete loss of mono-ubiquitination is R188S. (**F**) Protein sequence comparison of RhoA and RhoC. Shown are functional domains, positions of ubiquitinated sites, and residues mutagenized in the study. Residues in red indicate RhoGDI (geranyl–geranyl) binding consensus motifs. Areas of effector binding indicated with red bar. R188 essential for LNX1 recognition indicated with a blue box. (**G**) K162 identified in this study as target of LNX1 is conserved with K147 in Ras proteins, where it has been shown to undergo regulatory NDU resulting in Ras activation^8^.

While we did not find evidence of SUMOylation (Figure S1) of endogenous Rho protein, mono-, di- and tri-ubiquitination are readily detectable in cells and tissue (Fig. 1A). Pull down using the ubiquitin interacting domain followed by immunoblot analysis with antibody detecting all three RhoA/B/C proteins showed identical patterns of NDU in whole mouse brain and human HEK293T cells. In addition, mono-ubiquitinated RhoC is present in human skin fibroblasts and H1 human embryonic stem cells (Figure S7). This suggests that Rho protein NDU is widespread, rather than tissue or cell type specific. Rho proteins are known to undergo polyubiquitination induced degradation (PUD). In addition, RhoC has been shown to interact with E3 ubiquitin ligase, LNX1^9^. We asked whether overexpression of Smurf1, a principal E3 ligase responsible for Rho protein PUD^10^, LNX1 or closely related LNX2 can induce RhoC NDU. Co-transfection of HEK293T cells with 3xHA-RhoC and LNX1, LNX2 or Smurf1 followed by HA co-immunoprecipitation (IP), revealed robust monoubiquitination by LNX1, and to a lesser degree by LNX2 (Fig. 1B and S1B). However, Smurf1 transfected cells showed no increase in NDU compared to controls (Fig. 1B). Whether LNX1 also targets other members of the Rho family was tested. Co-expression of HA-tagged Rho GTPases with LNX1 in HEK293T cells resulted in robust monoubiquitination only of RhoC, and, to a lesser degree, of Cdc42 (Fig. 1C). Surprisingly, RhoA was not targeted by LNX1. This was unexpected, as RhoA and RhoC are 92% identical at the amino acid sequence level (Fig. 1F). Therefore, the lysine residues that are targeted by LNX1 were sought. We found that either a dual substitution of K133 and K135 for R or mutating K162 with R results in a substantial reduction of monoubiquitinated RhoC (Fig. 1D). However, the amino acids in the immediate vicinities of K133/135 and K162 are identical between RhoA and RhoC and thus could not explain LNX1 specificity for RhoC over RhoA (Fig. 1F). We therefore examined a selection of sites that significantly differ between RhoA and RhoC. Interestingly, mutation of a single residue, R188, to a serine found in the homologous position in RhoA, nearly completely abolished monoubiquitination of RhoC by LNX1 (Fig. 1E).

**Figure 2:**
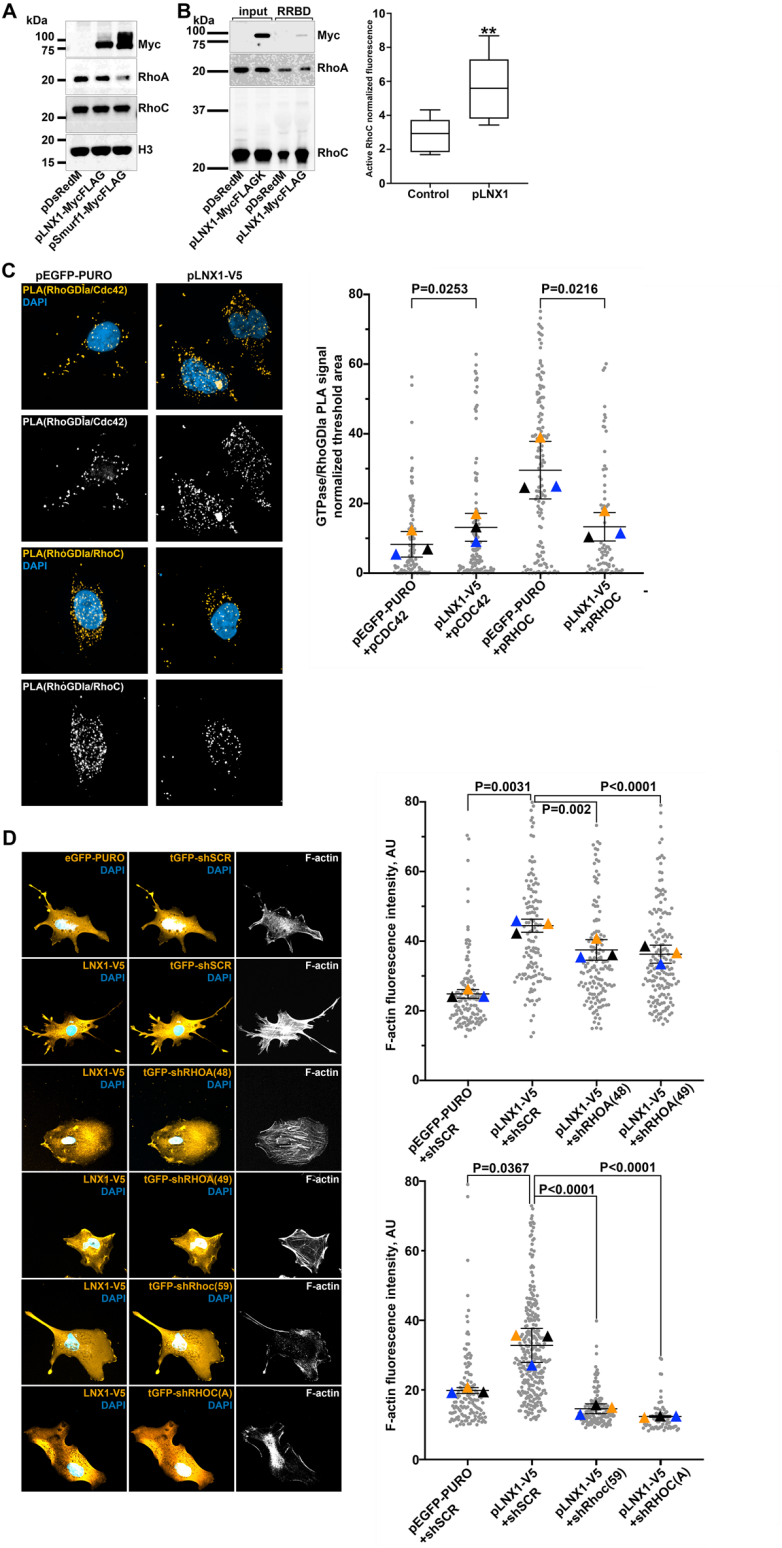
LNX1 activates RhoC by inhibiting its interaction with RhoGDIα. (**A**) Western blot analysis of transfected HEK293T cells expressing DsRedM (control), Smurf1-MycFLAG or LNX1-MycFLAG proteins. Immunoblot with Myc shows overexpression of Smurf1 or LNX1. Immunoblot for RhoA shows reduction in RhoA upon Smurf1 overexpression. Immunoblot for RhoC shows that LNX1 overexpression does not affect endogenous RhoC protein level. H3 is a loading control. (**B**) Rhotekin Rho Binding Domain (RRBD) pull down from transfected HEK293T cells expressing DsRedM (control) or LNX1-MycFLAG protein. LNX1 overexpression results in significant increase in the amount of endogenous RhoC pulled down by RRBD reflecting increase in the amount of GTP-bound (active) form of RhoC. Graph quantifies results from *n* = 5 biologically independent experiments. **P = 0.0037. Data are presented as the mean ± s.d. (**C**) PLA in 293-CDC42 control or LNX1-overexpressing cells using HA/RhoGDIα or RhoC/RhoGDIα probes. DAPI (blue), PLA signal (orange). Scale bar, 10 μm. Graph quantifies results from three biologically independent experiments. Data are presented as individual data points in gray circles and their mean ± s.d. within each of 3 independent biological replicates as triangles color coded by experiment. (**D**) Filamentous actin content (phalloidin, white) of ReN cells transduced with a lentivirus to overexpress LNX1-V5 with or without RhoA or RhoC knock-down. Immunolabels with anti-eGFP, anti-tGFP or anti-V5 (orange) identify transduced cells. Scale bar, 10 μm. Graphs are presented as individual data points from all three biological replicates in gray circles and their mean ± s.d. as triangles color coded by experiment. All P values were obtained using a two-tailed paired t-test.

Most often, protein ubiquitination by E3 ligases results in proteasomal degradation. We therefore investigated whether this is also the case for LNX1 and RhoC. PUD induced through overexpression of Smurf1 caused visible reduction in RhoA protein levels, consistent with previous reports^10,11^, but not RhoC (Fig. 2A). However, no such reductions in either RhoA or RhoC total protein levels were observed upon LNX1 overexpression (Fig. 2A). Therefore, the functional significance of LNX1 ubiquitination of RhoC must be something other than degradation. Among Ras proteins, NDU results in activation of those GTPases^8^. We therefore measured activity of Rho proteins using a classic approach that employs pull down with Rhotekin Rho binding domain (RRBD) peptide, which only interacts with GTP-loaded (active) RhoA and RhoC. Consistent with the role of NDU in Ras proteins and the fact that a fraction of the target protein is monoubiquitinated^8^, LNX1 overexpression resulted in a significant increase (197% of control, P < 0.004) in the amount of active total RhoC pulled down by RRBD (Fig. 2B). Central in the control of Rho proteins is Rho GDP Dissociation Inhibitor (RhoGDI), which prevents their activation by inhibiting GDP to GTP exchange and extraction of Rho from the plasma membrane^12^. Interaction between RhoGDI and Rho proteins is regulated by post-translational modifications^12^, therefore we asked if RhoC NDU-associated LNX1 overexpression affects RhoC interaction with RhoGDI. Using a Proximity Ligation Assay (PLA)^13^, we quantified RhoC binding to RhoGDIα. In this assay, antibodies specific for two proteins of interest are bound by fluorescently labeled aptamers that can only be ligated and amplified, generating signal, if the target proteins are located within 40 nm of each other. A significant decrease in the PLA signal was evident upon LNX1 overexpression, indicative of reduced RhoC-RhoGDIα interaction (Fig. 2C). In contrast, Cdc42-RhoGDIα interaction was slightly, but significantly elevated (Fig. 2C). This is consistent with Rho GTPase crosstalk at the level of RhoGDIα binding^12,14^. Visible reduction in the amount of RhoGDIα co-immunoprecipitated with RhoC was also evident when wild type RhoC was co-overexpressed with LNX1 (Figure S2). This difference was completely abolished by a RHOC^R188S^ mutation that prevented RHOC NDU (Figure S2). The functional consequence of LNX1 mediated RhoC NDU was examined in a human neuroepithelial stem cell line, ReN. A hallmark of Rho protein activation is formation of actin stress fibers^15^. Consistent with biochemical data, LNX1 overexpression also induced stress fiber formation in these cells (Fig. 2D). This was partially blocked by RhoA and completely blocked by RhoC knock-down (Fig. 2D), indicating that LNX1 acts upstream of RhoC in stress fiber formation. Taken together, these data demonstrate that RhoC NDU by LNX1 led to inhibition of the GTPase interaction with RhoGDIα, which was associated with total RhoC activation. Enhanced RhoC activity translates into physiological effects such as formation of stress fibers.

**Figure 3:**
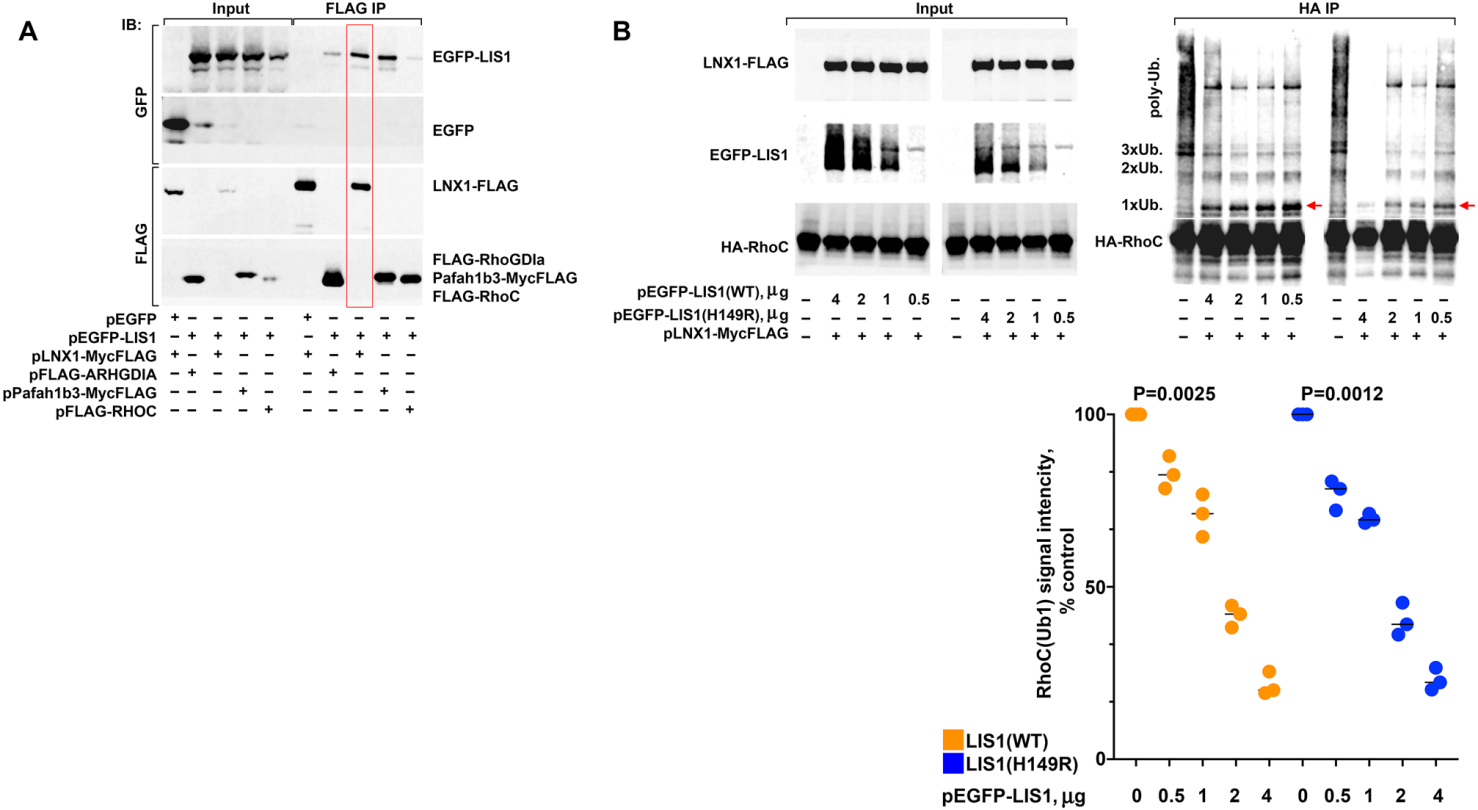
Lis1 binds to LNX1 and inhibits RhoC monoubiquitination. (**A**) Lis1 co-immunoprecipitates with LNX1. Anti-FLAG pull-down from HEK293T cells co-transfected with pEGFP-LIS1 and pPafah1b3-MycFLAG (positive control). EGFP-LIS1 co-immunoprecipitated with pLNX1-MycFLAG, but not pFLAG-ARHGDIA or pFLAG-RHOC. EGFP alone (negative control) does not co-immunoprecipitate with LNX1-MycFLAG. Therefore, we confirm Lis1-Pafah1b3 binding, identify novel Lis1-LNX1 interaction and show lack of direct interaction between Lis1 and RhoGDIα or RhoC. (**B**) Lis1 inhibits LNX1 mediated RhoC monoubiquitination in a dose-dependent manner. HA co-immunoprecipitation from HEK293T cells co-transfected with pcDNA3.1(+)-3xHA-RHOC, pLNX1-MycFLAG and varying amounts of pEGFP-LIS1(WT). Graph quantifies results from three independent experiments. Data are expressed as % control, where control is set at 100% and mean values are presented. P values were obtained using a two-tailed paired t-test.

The LNX family is distinguished among E3 ligases by the presence of four PDZ domains^16^. PDZ domains function, via protein–protein interactions^17^, to modulate cellular signaling events that include regulation of enzymatic activity^18^. We hypothesized that the LNX1-RHOC pathway is regulated by LNX1 protein–protein interactions. High throughput analysis of LNX1 binding proteins previously identified platelet activating factor acetylhydrolase subunit 1b3 (PAFAH1B3)^19^ and nuclear distribution E homolog like 1 (NUDEL)^20^ as LNX1 interactors. Another PAFAH subunit, 1B1 (aka LIS1), is mutated in a human brain malformation called lissencephaly (smooth brain), causing neuronal migration abnormalities associated with LIS1 haploinsufficiency^21,22^. Both PAFAH1B3^23^ and NUDEL^24^ bind LIS1, and we previously demonstrated a role for LIS1 in regulating activity of Rho proteins^25^. However, the molecular mechanism that links LIS1 levels to the activity of Rho proteins remains unknown. In order to explore the possible connection of LIS1 to the LNX1-RHOC pathway, we tested whether LIS1 binds to LNX1. In HEK293T cells co-transfected with EGFP-Lis1 and LNX1-MycFLAG, anti-FLAG beads readily co-immunoprecipitated LNX1 and LIS1 (Fig. 3A). Deletion and truncation mutations in *Pafah1b1* invariably reduce the total level of its encoded, LIS1, protein^26^. This is also true of at least some missense mutations that destabilize and consequently reduce LIS1 protein levels^27^ (Figure S3). The severity of LIS1 deficient phenotypes is sensitive to LIS1 protein levels, such that absence of LIS1 in *Lis1−/−* mutant mice produces embryonic lethality, and *Lis1*+*/–* haploinsufficiency (50% LIS1 reduction) produces hippocampal disorganization. While presence of the flox sites in the absence of Cre recombinase (*Lis1fl/fl*) slightly reduces LIS1 levels without obvious phenotype, a biallelic *Lis1fl/–* genotype produces viable neonate pups compromised by 60–75% LIS1 reduction and marked cerebellar and cortical disruption^21^. Therefore, we hypothesized that any effect of LIS1 on LNX1-RhoC might be LIS1 dose-dependent. Indeed, gradual increase in LIS1 overexpression correlated with a gradual decrease in monoubiquitinated RhoC (Fig. 3B). At the same time, the H149R mutation in LIS1 that causes the most severe lissencephaly phenotype among missense mutations, destabilizes LIS1 to reduce its level (Figure S3), but does not disrupt the ability of LIS1 to bind LNX1 (Figure S4). The data suggest that pathological activation of LNX1 and RhoC contributions to a lissencephaly phenotype may be the consequence of reduced LIS1 protein levels rather than missense-dependent disruption of LNX1-LIS1 specific binding.

**Figure 4:**
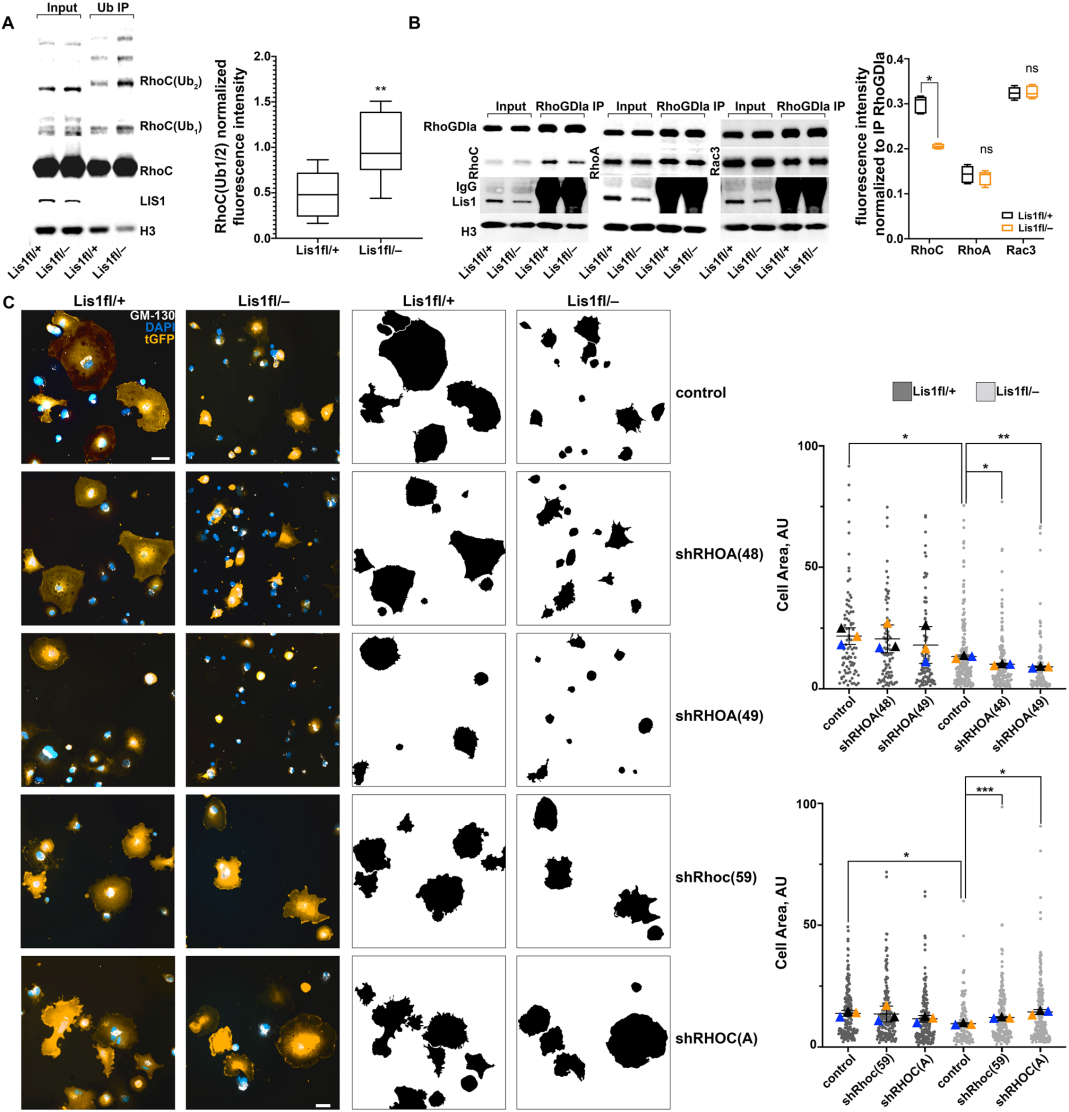
Reduced level of Lis1 specifically activates RhoC in brain. (**A**) Abnormal increase in RhoC NDU in *Lis1*^*fl/−*^ cortex. Ubiquitin binding domain pull down from *Lis1*^*fl/*+^ or *Lis1*^*fl/−*^ brain is increased in mono-, di- and tri-ubiquitinated RhoC when Lis1 protein is reduced. Graph quantifies results from *n* = 5 biologically independent experiments, **P = 0.0037. Data are shown as the mean ± s.d. (**B**) Elevated RhoC NDU disrupts its interaction of total RhoC with RhoGDIα in vivo. RhoGDIα immunoprecipitation from *Lis1*^*fl/*+^ or *Lis1*^*fl/−*^ brain shows decreased RhoC binding, while RhoA and Rac3 remain unchanged. Graph quantifies results from *n* = 3 biologically independent experiments done in duplicate, *P < 0.0312, ns = not significant with *P* = 0.4375 (RhoA) and *P* < 0.9999 (Rac3). Data are shown as the mean ± s.d. (**C**) RhoC, but not RhoA knock down by shRNAi rescues cell spreading impairment in *Lis1*^*fl/−*^ neonatal astrocytes. Immunostaining of *Lis1*^*fl/*+^ or *Lis1*^*fl/−*^ astrocytes transduced with a GFP-expressing lentivirus to knock down RhoA or RhoC for tGFP (anti-tGFP, orange). Golgi apparatus (GM-130, white) and DNA (DAPI, blue). GFP staining is used to define cell area (shown in the black and white mask of transduced cells) measured in arbitrary units (AU) in Image J. Control = scrambled shRNA transduced cells. Scale bar, 10 μm. Graph represents cell area measurements from three independent experiments. Data are presented as individual data points from all three biological replicates in dark gray (*Lis1fl/*+) or light gray (*Lis1fl/–*) circles and their mean ± s.d. as triangles color coded by experiment. *P < 0.05, **P < 0.01, ***P < 0.001. Where indicated, statistical significances were obtained using a two-tailed paired t-test for amount of ubiquitinated RhoC (**A**) and cell spreading assay (**C**), or non-parametric, two-tailed Wilcoxon matched-rank signed test for the RhoGDIα IP (**B**).

Since overexpression of LIS1 inhibits LNX1 and reduces RhoC NDU in HEK293T cells (Fig. 3), we reasoned that levels of RhoC NDU would be elevated in the *Lis1fl/–* mouse model of lissencephaly^21^. We estimate that LIS1 levels in the brain of newborn *Lis1fl/*– animals are reduced by about 60% compared to *Lis1*+*/*+ controls (Fig. 4A), and this is accompanied by a robust, close to + 100% (p < 0.005) increase in mono-, di- and tri-ubiquitinated RhoC in the *Lis1fl/–* brain (Fig. 4A). Since LNX1 overexpression in HEK293T cells and consequent increases in RhoC NDU results in attenuated binding of RhoC to RhoGDIα (Fig. 2), we examined *Lis1fl/–* brain to determine whether physiologically relevant reduction in LIS1 levels also decreases RhoC binding to RhoGDIα in vivo, presumably via increased LNX1 E3 ligase activity. Indeed, immunoprecipitation of RhoGDIα from *Lis1fl/–* brain pulled down less RhoC, while RhoA and Rac3 were unchanged (Fig. 4B). Collectively, our biochemical data points to a specific upregulation of RhoC, but not RhoA activity upon reduction in LIS1 level. Cellular consequences of abnormal RhoC activation, including actin stress fiber formation, are largely indistinguishable from that of RhoA^28–30^. However, we hypothesize that in the context of reduced LIS1 level, targeted reduction of RhoC activity should have a greater rescue effect than RhoA on cell morphology. To test this hypothesis, we utilized a cell spreading assay in primary astrocytes that were isolated from the brains of *Lis1fl/*+ or *Lis1fl/–* neonatal mice. Efficient cell spreading requires temporary reduction in Rho protein activity, achieved via tyrosine phosphorylation and activation of p190-RhoGAP downstream of integrin receptor signaling^31^. Hence, elevated activity in RhoC is expected to prevent normal integrin dependent spreading. In this assay, trypsin treated normal cells re-plated onto laminin substratum rapidly spread, while impairment in spreading keeps cells rounded up and this can be easily quantified as reduction in cell area. Indeed, *Lis1fl/–* astrocytes displayed significantly reduced cell area 1 h. after replating onto laminin compared to *Lis1fl/*+ cells (Fig. 4C). Interestingly, RNA interference using shRNA against RhoA further magnified the cell spreading impairment (Fig. 4C). In contrast, and consistent with our hypothesis, RhoC knock-down resulted in a significant increase in cell area (Fig. 4C), indicating differential actions of downstream effectors of these Rho proteins^32^.

## Discussion

Unlike polyubiquitination that typically leads to proteasomal degradation, mono-, di-, or tri-ubiquitination can regulate target protein function without altering protein levels^33–35^. The present study has demonstrated a previously unknown signaling module in which LNX1 selectively monoubiquitinates RhoC and leads to activation of total RhoC. Data provided here suggest that at least one mechanism of this RhoC activation occurs through inhibition of total RhoC-RhoGDIα interaction that is associated with NDU of RhoC. The molecular mechanism by which a subfraction of monoubiquitinated proteins exert outsized effects on the total target protein is unknown, though it is widely observed^8,36–40^. Our results argue against the possibility that LNX1 modulates a GEF, GAP or effector downstream target that indirectly leads to the activation of total RhoC, since RhoC and RhoA share the vast majority of their GAPs, GEFs and effectors. If LNX1 acted on any of those, it would also activate RhoA, which it does not, as we show. The RhoC and/or RhoA specific GAPs/GEFs that have been proposed in the literature are very few, their expression is restricted, and would not account for widespread activation of RhoC. Potential mechanisms for this outsized effect include that monoubiquitinated RhoC itself interacts with total non-ubiquitinated RhoC to block its interaction with RhoGDIα or that monoubiquitinated RhoC has some as yet unidentified downstream effector that amplifies signaling through a RhoC-specific GEF or GAP. Thus, the direct mechanism of action in the association of RhoC NDU with total RhoC activation remains to be determined.

Along with LNX1-dependent NDU of RhoC, we identify down-regulation of this interaction by LIS1 suppression of LNX1 mediated NDU. These molecular relationships were extensively validated using gene-dosage, protein–protein interaction, activity assays, mutagenesis, cell biological and cell phenotype rescue studies. Results show that: 1. LNX1 mediates RhoC NDU. Further, this is specific to RhoC, and not RhoA, and we identify which amino acid residues in RhoC are involved; 2. Monoubiquitination renders total RhoC more active, associated with the disruption of RhoC interaction with RhoGDIα. This is shown on both a biochemical activity assay and a cell biological read out of RhoC activity; 3. LIS1 directly binds to LNX1 and, in a dose-dependent manner, inhibits LNX1 dependent NDU of RhoC; 4. Consequently, reduced LIS1 results in enhanced RhoC NDU and increases total RhoC activity in cells and whole brain tissue; 5. Finally, aspects of the LIS1 deficiency cellular phenotype that are dependent on enhanced RhoC activity can be rescued by RhoC knock-down.

These data hold relevance for both translational and basic cell biological research. From the basic biology point of view, we expand the role of NDU in the Ras superfamily to include Rho-GTPases. Interestingly, the Lysine that undergoes NDU in RhoC is conserved with previously reported monoubiquitination sites in H-, N- and K-Ras^8^ (Fig. 1G). Our mutagenesis data provide a foundation for structural characterization of LNX1-RhoC recognition and illuminates the ability of LNX1 to distinguish between RhoA and RhoC that are over 90% identical at the amino acid sequence level. Furthermore, this study provides the first molecular mechanism to account for the profound effect of LIS1 on Rho proteins^25,41–45^. LIS1 directly binds and inhibits LNX1. Domains and residues involved in LIS1-LNX1 binding are yet to be characterized and this will undoubtedly aid in designing LNX1 inhibitors.

Results reported here also indicate that Cdc42 is a target of LNX1. We previously reported the elevation of RhoA/C activity in LIS1 deficient neurons accompanied by concomitant reductions in Rac1 and Cdc42 activity^25,41^, and that Rac1 and Cdc42 activity could be rescued by ROCK inhibitor indicating Rho GTPase crosstalk as an important mechanism^25^. This crosstalk is corroborated in the present study by the demonstration of decreased RhoC-RhoGDIα binding and increased Cdc42-RhoGDIα binding that would increase RhoC-GTPase activity and decrease Cdc42 activity. Thus, at least three mechanisms account for down regulation of Cdc42/Rac1 when LIS1 levels are reduced: (1) ROCK inhibition of Rac1/Cdc42^46,47^; (2) reduction in perimembrane IQGAP1^41^; and (3) increased binding to RhoGDIα (Fig. 2C). It is possible that additional targets of LNX1, in particular Rho GAP and/or Rho GEF proteins, may also result in altered Rho GTPase activity. However, unlike the hundreds of GEF and GAP proteins, there are only three GDIs. Only GDIα, used here, is expressed in all cells, while the other two are very restricted in their expression. For that reason alone, this regulatory mechanism is most probably of central importance to RhoC regulation in a broad swath of cell types and physiological contexts.

Since LNX1 and RhoC expression is exclusive to vertebrates, this LIS1-LNX1-RhoC module is an evolutionarily new function of highly conserved LIS1. Our data provide a rare example of Rho isoform specific modulation upstream of these essential GTPases. Thus, this study opens new opportunities for therapeutic intervention relevant to numerous human diseases in which GTPase activities are disrupted, including cancers, vascular disease, neurodevelopmental and neurodegenerative diseases. For example, LNX1 promotes proliferation of neural stem cells^48^ and is a glioblastoma stemness factor^49^, RhoC is indispensable for tumor progression into advanced metastatic stages^50^, and both LIS1^51^ and LNX1^52^ are hubs underpinning genetic networks disrupted in multiple neurological diseases. RhoC has been proposed as a candidate therapeutic target in conditions including cancer^50^, neurodevelopmental^53^ and neurodegenerative^54,55^ diseases, though options for selective manipulation are lacking in view of its high degree of homology with RhoA. Thus, identification of LNX1 as an upstream activating molecule presents an intriguing therapeutic opportunity, in which inhibition of LNX1 is expected to reduce activity of RhoC, but not RhoA.

## Methods

All required regulatory and ethical approvals for the laboratory methods and procedures used in this study were obtained from Weill Cornell Medicine, including review and approval from the Institutional Animal Care and Use Committee (IACUC), which is accredited by the American Association for the Accreditation of Laboratory Animal Care International (AAALAC). Methods employed for use of laboratory animals in the study comply with the policies of the U.S. Public Health Service and are reported in accordance with ARRIVE guidelines (https://arriveguidelines.org).

### Mice

129S-*Pafah1b1*^*tm2Awb*^/J mice that possess loxP sites flanking exons 3 through 6 of the *Pafah1b1 (Lis1*^*fl*^*)* gene were obtained from The Jackson Laboratory (stock #008002). *Pafah1b1*^*Neo*^ (*Lis1*^−^) mouse line where exon 6 of the *Pafah1b1* gene is replaced with PGK-Neo cassette was generously provided by Dr. Anthony Wynshaw-Boris. All animal usage, maintenance and experimental protocols were approved by the Institutional Animal Care and Use Committee of Weill Cornell Medicine. Complete loss of *Pafah1b1* gene expression results in early embryonic lethality, therefore *Lis1*^+*/−*^ mice were maintained as heterozygotes. *Lis1*^*fl/−*^ compound heterozygous mice were generated by crossing *Lis1*^+*/−*^ heterozygous and *Lis1*^*fl/fl*^ homozygous animals. In the resulting *Lis1*^*fl/−*^ compound heterozygous mice one *Pafah1b1* allele is null (exon 6 is replaced with PGK-Neo) and the other *Pafah1b1* allele contains loxP sites that flank exons 3 through 6. Presence of loxP sites slightly reduces expression of *Pafah1b1* gene, therefore *Pafah1b1* gene dosage in *Lis1*^*fl/−*^ animals is somewhere in between *Lis1*^+*/−*^ heterozygous and *Lis1*^*−/−*^ homozygous animals^21^.

### Western blot analysis

Postnatal day 1 (P1) whole brain or cell culture lysates were prepared in RIPA buffer (Thermo Fisher, 89901) supplemented with 20 mM NEM (Sigma, E3876), 1 × protease inhibitor cocktail (Thermo Fisher, 1861279), 1 × phosphatase inhibitor cocktail (Sigma, P5726) and 100 U/ml UNase (Thermo Fisher, 88701). Protein concentrations were determined using a BCA assay per manufacturer’s protocol (Thermo Fisher, 23225). 10–15 μg total protein per well were separated on pre-cast Bis–Tris gels at 200 V for 35–40 min, then transferred onto 0.2 μm nitrocellulose membrane (Bio-Rad, 1620146). After transfer, when the antibodies being used were incompatible for dual labeling on the intact blot, the broad area (based on protein standards on the blot) that encompassed the molecular weight of the protein(s) of interest was cut horizontally across the membrane for separate immunostaining. In all cases, whether intact or reassembled, the entire vertical extent of the membrane was digitally imaged on the Odyssey digital imager, to ensure proper identification of the apparent molecular weight of the protein being detected. All antibodies used have been extensively studied and their specificity for the target antigen is well established (Table 1). Membranes were treated with antibody extender solution (Thermo Fisher, 32110). Membranes were blocked and all antibody dilutions were made in Odyssey blocking buffer (Li-COR, 927-50000) supplemented with 0.05% Tween 20 (Sigma, P1379). Primary antibody incubations were carried out overnight at + 4 °C before incubations in secondary antibody (Table 2) for 1 h. at room temperature. Protein bands were visualized, and band intensities measured on an Odyssey Fc machine (Li-COR). Western blot data acquisition and analysis were performed with ImageStudio 2 software (Li-COR). Full blots used to prepare figures are provided in supplementary material.

**Table 1:** Primary antibodies for immunostaining, western blotting and proximity ligation assay.

Antibody target	Manufacturer	Catalogue #	Western blot dilution	Immunofluorescence and PLA dilution
Beta-actin	Sigma	A1978	1:5,000	
RhoA/B/C	Millipore	05-822	1:500	
H3	Cell Signaling	14269	1:5,000	
Myc	Bethyl Labs	A190-104A	1:1,000	
HA	Bethyl Labs	A190-107A	1:1,000	
HA	BioLegend	901502		1:4,000
DDK (a.k.a. FLAG)	Cell Signaling	14793	1:5,000	
RhoA	Cell Signaling	2117	1:5,000	
RhoC	Cell Signaling	3430	1:5,000	
RhoC	OriGene	TA806448		1:4,000
RhoGDIα	Santa-Cruz Biotechnology	13120	1:5,000	
RhoGDIα	Cell Signaling	2564		1:200
COX4	Cell Signaling	4850	1:1,000	
EGFP	Rockland	600-101-215	1:1,000	1:1,000
V5	Bethyl Labs	A190-119A		1:500
Lis1	Sigma	L7391	1:5,000	
NUDEL	Abcam	ab124895	1:5,000	
Rac3	Abcam	ab129062	1:5,000	
Ubiquitin	Cell Signaling	3936	1:1,000	
SUMO1	Santa-Cruz Biotechnology	5308	1:500	
SUMO2/3/4	Santa-Cruz Biotechnology	393144	1:500

**Table 2:** Secondary antibodies for immunostaining, western blotting and proximity ligation assay.

Antibody name/Conjugate	Manufacturer, catalogue #	Western blot dilution	Immunofluorescence dilution
Donkey anti-mouse AlexaFluor Plus 555	Thermo Fisher, A32773		1:1,000
Donkey anti-goat AlexaFluor 488	Thermo Fisher, A11055		1:1,000
Donkey anti-goat AlexaFluor 647	Thermo Fisher, A31573		1:1,000
Donkey anti-mouse IRDye 800CW	LI-COR 926-32212	1:10,000	
Donkey anti-goat IRDye 680LT	LI-COR 926-68024	1:10,000	
Donkey anti-rabbit IRDye 680RD	LI-COR 926-68073	1:10,000	
Donkey anti-rabbit IRDye 800CW	LI-COR 926-32213	1:10,000	

### Pull down and Co-IP based assays

Ubiquitinated proteins were pulled down from whole tissue or cell lysates using a SignalSeeker Ubiquitination Detection kit (Cytoskeleton, BK161), as per manufacturer’s protocol. HA-tagged and interacting proteins were pulled down from cell lysates using a Pierce HA-Tag Magnetic IP/Co-IP Kit (Thermo Fisher, 88838) following the manufacturer’s protocol. Active Rho GTPases were pulled down from cell lysates using RhoA pull-down Activation Assay Biochem Kit (Cytoskeleton, BK036), as per manufacturer’s protocol. FLAG-tagged and interacting proteins were pulled down from cell lysates using EZview Red Anti-FLAG M2 Affinity Gel (Sigma, F2426), as per manufacturer’s protocol. For RhoGDIα pull down, cells were lysed in CelLyticM reagent (Sigma C2978) supplemented with 20 mM NEM (Sigma, E3876), 1 × protease inhibitor cocktail (Thermo Fisher, 1861279), 1 × phosphatase inhibitor cocktail (Sigma, P5726) and 100 U/ml UNase (Thermo Fisher, 88701). Lysates were clarified by centrifugation at 18,000*g* for 1 min at room temperature. Supernatants were transferred to clean Eppenforf tubes and incubated with agarose conjugated mouse anti-RhoGDIα antibody (Santa-Crus Biotechnology, SC-373724 AC) overnight at + 4 °C. After overnight incubation agarose was briefly spun down and washed three times with CelLyticM reagent. Proteins bound to agarose were eluted by incubating in 1 × gel loading buffer (LI-COR, 928-4004) for 10 min at + 70 °C. Input, pull-down and co-IP samples were subjected to Western blot analysis as above.

### Immunofluorescence

Cells were fixed with 3.7% paraformaldehyde pre-warmed to + 37 °C for 10 min at room temperature. After fixation, cells were rinsed 3 × with water, then blocked with 0.5% BSA (Millipore, 2930) + 1% fish gelatin (Sigma, G7041) + 1% donkey serum (Jackson ImmunoResearch, 017-000-121) in PBS and permeabilized with 0.2% Triton × 100. All antibody dilutions were made in blocking buffer. Primary antibody (Table 1) incubations were overnight at + 4 °C and secondary (Table 2) 1 h. at room temperature. After secondary antibody incubation cells were counterstained with DAPI and mounted in ProLong Gold antifade reagent (Thermo Fisher, P36934). Images were collected on Zeiss Axiovert 200 M inverted microscope fitted with spinning disk confocal head (Perkin-Elmer) and Borealis illumination (Andor Technology). All image data analysis was performed using Fiji software.

### Proximity ligation assay

Cells were fixed with 3.7% paraformaldehyde pre-warmed to + 37 °C for 10 min at room temperature, then rinsed 3 × with water, blocked with 0.5% BSA (Millipore, 2930) + 1% fish gelatin (Sigma, G7041) + 1% donkey serum (Jackson ImmunoResearch, 017-000-121) in PBS and permeabilized with 0.2% Triton × 100. Following permeabilization, PLA was carried out using a MilliporeSigma Duolink^®^ In Situ Orange Starter Kit Mouse/Rabbit (cat. # DUO92102), as per manufacturer’s protocol. Available RhoA or Cdc42 specific antibodies proved unsuitable for staining. Therefore, to compare RhoC to a different member of Rho family we used 293-CDC42 cells that stably express HA-tagged Cdc42. The specificity of RhoGDIα (Cell Signaling cat. # 2564) and anti-HA antibody (BioLegend cat. # 901502) were validated by the manufacturer. Anti-RhoC (OriGene cat. # TA806448) was validated using RhoC knocked down cells (Figures S5 and S6).

### Site-directed mutagenesis and molecular cloning

EGFP-tagged SUMO vector was prepared by transferring the SUMO1 open reading frame (ORF) from pSG5-His-SUMO to pEGFP-C1 using BamHI sites and standard molecular cloning methods. For mutagenesis, RHOC ORF was transferred from pcDNA3.1(+)-3xHA-RHOC to pBlueScript using KpnI/DraIII sites and standard molecular cloning methods. All mutagenesis reactions were done in pBlueScript vector. Introduction of point mutations into RHOC ORF was carried out using the QuickChange II Site-Directed Mutagenesis Kit (Agilent Technologies cat. # 200521) with primers listed in Table 3, as per manufacturer’s protocol. After mutagenesis, mutant RHOC was transferred back to pcDNA3.1(+)-3xHA-RHOC mammalian expression vector and constructs confirmed by Sanger sequencing. The 3xHA-RHOC ORF was transferred from pcDNA3.1(+)-3xHA-RHOC to pCMV-Tag2 using HindIII/XhoI sites and standard molecular cloning methods. The DNA sequence validation of all final constructs was performed using the Sanger sequencing method.

**Table 3:** Primers for mutagenesis.

Mutagenesis primers		Amino acid change
Forward	GGCAGAAGTGCCTCACCTCTGGGGTCC	K104R
Reverse	GGACCCCAGAGGTGAGGCACTTCTGCC	
Forward	CTCGTCTTGCCTCAGGTCTAGCCTATTCCCCACCAGGATGATGGG	K118R, K119R
Reverse	CCCATCATCCTGGTGGGGAATAGGCTAGACCTGAGGCAAGACGAG	
Forward	CGGGCTCCTGCCTCATCCTGGCCAGCTCTCT	K133R, K135R
Reverse	AGAGAGCTGGCCAGGATGAGGCAGGAGCCCG	
Forward	GTCTTGGCTGAGCACTCCATGTAGCCAAAGGCACTGA	L157M
Reverse	TCAGTGCCTTTGGCTACATGGAGTGCTCAGCCAAGAC	
Forward	CTCCCTCCTTGGTCCTGGCTGAGCACTCA	K162R
Reverse	TGAGTGCTCAGCCAGGACCAAGGAGGGAG	
Forward	CTTGTTCTTGCGGGCCTGGAGGCCAGC	V181A
Reverse	GCTGGCCTCCAGGCCCGCAAGAACAAG	
Forward	CCGACGCTTGTTCCTGCGGACCTGGAG	K183R
Reverse	CTCCAGGTCCGCAGGAACAAGCGTCGG	
Forward	GAATGGGACAGCCGCTCCGACGCTTGT	R188S
Reverse	ACAAGCGTCGGAGCGGCTGTCCCATTC	
Forward	GGCCTTCCTCAGGCCGAACGGGCTC	S141P
Reverse	GAGCCCGTTCGGCCTGAGGAAGGCC	

### Lentiviral production and transduction

Lentiviral particles were prepared according to Broad Institute’s The RNAi Consortium (TRC) protocol: “Lentivirus production of shRNA, CRISPR, or ORF-pLX clones in 10 cm dishes or 6-well plates” with plasmids listed in Table 4. The following modification to the protocol was made. Transfection reagent used was PolyJet (SignaGen, SL100688). Pooled (24 and 72 h. collection) media supernatants were filtered through 0.45 μm cellulose acetate filter (VWR, 28145-479), aliquoted and stored at − 70 °C. Cells were plated the day before to reach 50–70% confluency on the day of transduction. Culturing media was aspirated and thawed filtered lentiviral supernatant applied to cover cells. Polybrene (Millipore, TR-1003-G) was added to final concentration 4 μg/ml. Cell were incubated with lentiviral particles for 6 h. under normal culturing conditions. After 6 h. incubation, supernatant was removed, and cells fed with their normal culturing media.

**Table 4:** Plasmids for transfection and lentivirus preparation.

Plasmid name	Protein	Manufacturer, catalogue #	Source
pDsRedM	DsRedM	TaKaRa, 632465	
pCMV6-LNX1-MycDDK (a.k.a. pCMV6-LNX1-MycFLAG)	LNX1-Myc-FLAG	OriGene, MR219190	
pCMV6-LNX2-MycDDK (a.k.a. pCMV6-LNX2-MycFLAG)	LNX2-Myc-FLAG	OriGene, MR217497	
pCMV6-Smurf1-MycDDK (a.k.a. pCMV6-Smurf1-MycFLAG)	Smurf1-Myc-FLAG	OriGene, MR210325	
pCMV6-PAFAH1B3-MycDDK (a.k.a. pCMV6-PAFAH1B3-MycFLAG)	PAFAH1B3-Myc-FLAG	OriGene, RC201268	
pcDNA3.1(+)-3xHA-CDC42	3xHA-CDC42	cDNA Resource Center, CDC420TN00	
pcDNA3.1(+)-3xHA-RAC1	3xHA-RAC1	cDNA Resource Center, RAC010TN00	
pcDNA3.1(+)-3xHA-RHOA	3xHA-RhoA	cDNA Resource Center, RHO0A0TN00	
pcDNA3.1(+)-3xHA-RHOC	3xHA-RhoC	cDNA Resource Center, RHO0C0TN00	
pMD2.G			Addgene, 12259
psPAX2			Addgene, 12260
pZIP-3xHA-RHOC(WT)-EGFP	3xHA-RhoC(WT), EGFP	Transomic technologies, custom order	
pZIP-3xHA-RHOC(R188S)-EGFP	3xHA-RhoC(R188S), EGFP	Transomic technologies, custom order	
pZIP-EGFP-PURO	EGFP-PAC	Transomic technologies, TLO2015	
pLX304-LNX1-V5	LNX1-V5	Horizon Discovery, OHS6085-213577001	
pEGFP-C1	EGFP	Clontech, discontinued	
pCMV-Tag2		Agilent Technologies, 211172	
pEGFP-LIS1	EGFP-LIS1		C.A. Walsh^56^
pGIPZ-shRHOA(48)		Horizon Discovery, RHS4430-200223832	
pGIPZ-shRHOA(49)		Horizon Discovery, RHS4430-200226019	
pGIPZ-shRhoc(59)		Horizon Discovery, RMM4431-200405470	
pGFP-C-shRHOC(A)		OriGene, TL302002A	
pSG5-His-SUMO			Addgene, 17271

### Cell culture

HEK293T (ATCC, CRL3216) were obtained from ATCC and maintained according to manufacturer’s instructions. ReN cell CX, a Human neural progenitor cell line, was obtained from MilliporeSigma (Sigma, SC007) and maintained according to manufacturer’s instructions. All cell lines were routinely maintained in antibiotic free media to monitor aseptic technique. Primary cultured astrocytes were prepared as follows. P1 cortices were dissected and put in PBS on ice. Tissue was transferred to 15 ml conical tubes and incubated in TrypLE (Thermo Fisher, 12605) for 30 min at + 37 °C while shaking at 50 rpm. After incubation, deoxyribonuclease I (Worthington Biochemical Corp., LS002007) 0.005% was added and tissue dissociated by trituration through a flame–drawn, narrow barrel tip Pasteur pipette fifty times. After trituration large pieces of remaining tissue were filtered out by passing through a 70 μm filter (Miltenyi Biotec, 130-098-462). Cells were spun down for 5 min at room temperature at 300 g. Supernatant was aspirated and cells resuspended in Astrocyte Media (Thermo Fisher, A1261301). Cultures were maintained in Astrocyte Media with fresh media exchanges every 3–4 days. Cells were passaged at 70% confluency and used in experiments at passage 2 and above, when cultures consisted exclusively of astrocyte cells as confirmed by immunostaining with GFAP. 293-CDC42 cells were prepared by transfecting HEK293T cells with pCMV6-3xHA-CDC42-PAC-mCherry and selected for stably transfected clones with 10 μg/ml Puromycin (ThermoFisher cat. # A1113803). Selection was monitored by expression of mCherry until 100% cells displayed mCherry fluorescence. Transient transfections of HEK293T cells were carried out using PolyJet (SignaGen, SL100688) reagent with plasmids in Table 4.

### Cell spreading assay

Primary astrocytes 7 days after transduction were dissociated with TrypLE (Life Technologies 12605). Trypsinized cell were spun down for 5 min at 300 g at room temperature. Supernatant was aspirated and cells resuspended in Astrocyte Media. Resuspended cells were plated onto poly-D-lysine (20 μg/ml, Sigma, P7280) and laminin (5 μg/ml, Thermo Fisher, 23017-015) coated glass coverslips in 12 well plate. Cells were incubated under normal culturing conditions for 1 h. After 1 h. incubation cells were fixed with 3.7% PFA.

### Statistical analysis and reproducibility

Arithmetic means and standard deviations were calculated and plotted in the graphs. The respective n values and statistical tests used are shown in figure legends. Statistical significances were obtained using tests specified and p values shown in figure legends.

Ubiquitin pull down followed by Western Blot (Figs. 1A and 4A), immunoprecipitation followed by Western Blot (Figs. 1B–E, 3A, B and 4B), Western Blot (Fig. 2A), Rhotekin pull down followed by Western Blot (Fig. 2B) experiments were performed from three to six times independently with similar results. Each experiment was run in duplicate with identical results. Proximity ligation assay (Fig. 2B), F-actin staining (Fig. 2C), and cell spreading assay (Fig. 4C) were performed three times independently with similar results.

## Supplementary Information

Supplementary Figures.

## Data Availability

All data supporting the findings of this study are available from the corresponding author on reasonable request. Any materials that can be shared will be released via a Material Transfer Agreement. The sources of all reagents used are given in Methods. Original Western blots are provided in Supplementary Data. Sequences for all constructs used are part of the publicly available data sources with their NCBI and Uniprot accession numbers listed in Table 5.

**Table 5:** Gene accession numbers.

Plasmid name	Gene name	NCBI accession number	Uniprot protein ID
pCMV6-LNX1-MycDDK (a.k.a. pCMV6-LNX1-MycFLAG)	*Lnx1*	NM_001159577.1	O70263-1
pCMV6-LNX2-MycDDK (a.k.a. pCMV6-LNX2-MycFLAG)	*Lnx2*	NM_080795.4	Q91XL2-1
pCMV6-Smurf1-MycDDK (a.k.a. pCMV6-Smurf1-MycFLAG)	*Smurf1*	NM_001038627.1	Q9CUN6-1
pcDNA3.1(+)-3xHA-CDC42	*CDC42*	NM_009861.3	P60766-2
pcDNA3.1(+)-3xHA-RAC1	*RAC1*	NM_006908.5	P63000-1
pcDNA3.1(+)-3xHA-RHOA	*RHOA*	NM_001313941.2	P61586-1
pcDNA3.1(+)-3xHA-RHOC	*RHOC*	NM_001042678.2	P08134-1
pEGFP-LIS1	*Pafah1b1*	NM_013625.4	P63005-1
pTag-ARHGDIA (a.k.a. pFLAG-ARHGDIA)	*ARHGDIA*	NM_001185077.3	P52565-1
pCMV6-Pafah1b3-MycDDK (a.k.a. pCMV6-Pafah1b3-MycFLAG)	*PAFAH1B3*	NM_001145939.2	Q15102-1 Gene accession numbers.
