# Supplementary material for: Activation of RhoC by regulatory ubiquitination is mediated by LNX1 and suppressed by LIS1
Source: Sci Rep. 2022 Oct 3;12:16493. doi: 10.1038/s41598-022-19740-1 (PMC9529947; doi:10.1038/s41598-022-19740-1)

Supplementary Figures

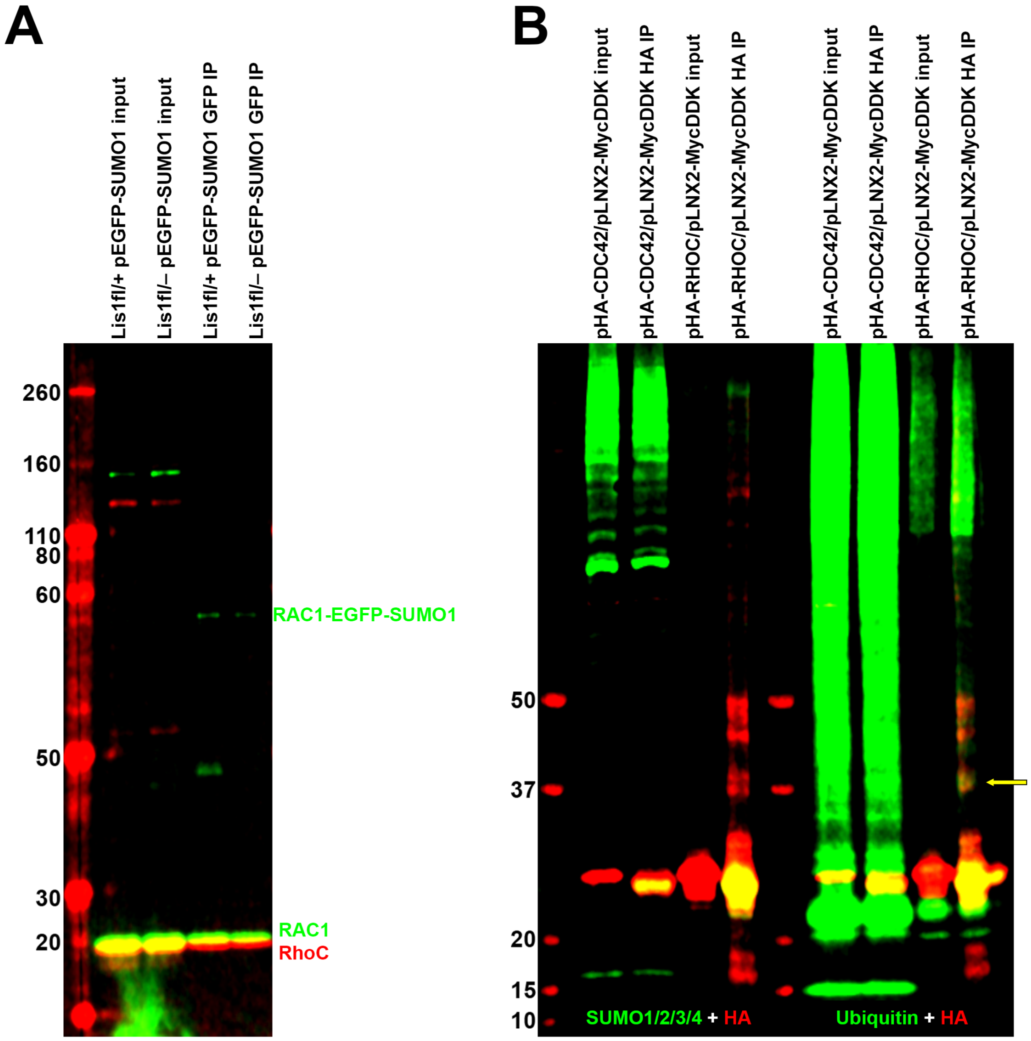

Figure S1: RhoC does not undergo post-translational SUMOylation.
a. GFP co-immunoprecipitation in SV40 transformed mouse neonatal fibroblasts extracted from *Lis1*$^{fl/+}$ or *Lis1*$^{fl/–}$ animals and transfected with pEGFP-SUMO1. Immunoblotting with Rac1 revealed three bands: 21 kDa unmodified Rac1, 41 kDa band corresponding in size to EGFP-SUMO1 and 62 kDa band corresponding in size to RAC1-EGFP-SUMO1. Immunoblotting with RhoC only revealed unmodified RhoC running at 20 kDa. **b**. HA co-immunoprecipitation in 293T cells co-transfected with pcDNA3.1(+)-3xHA-CDC42 + pCMV6-LNX2-MycDDK or pcDNA3.1(+)-3xHA-RHOC + pCMV6-LNX2-MycDDK. 3xHA-RhoC, but not 3xHA-CDC42 ran as multiple bands. Immunoblotting with a mixture of antibodies against SUMO1 + SUMO2/3/4 did not reveal any overlap with higher MW isoforms of RhoC. Immunoblot analysis with Ubiquitin revealed an overlap with 3xHA-RhoC marked by yellow arrow running at 34 kDa corresponding in size to monoubiquitinated 3xHA-RhoC. (Note: DDK encodes an amino acid sequence identical to FLAG peptide)

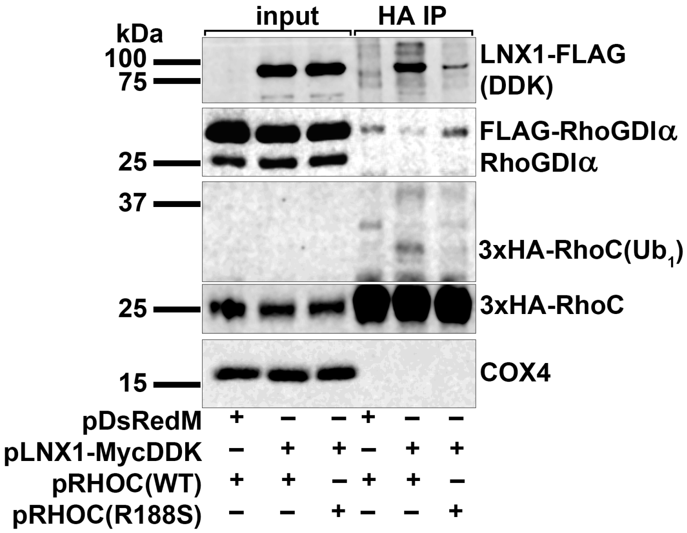

Figure S2: LNX1 activates RhoC by inhibiting its interaction with RhoGDIa. HA co-immunoprecipitation from lysates with added affinity-purified FLAG-RhoGDIa, from 293T cells that were co-transfected to express 3xHA-RHOC(WT or R188S) and DsRedM (negative control) or LNX1-MycDDK proteins. LNX1 overexpression results in the reduction in the amount of RhoGDIa pulled down with wild type (WT), but not R188S mutant RhoC. Three biologically independent experiments were performed with similar results. (Note: DDK encodes an amino acid sequence identical to FLAG peptide)

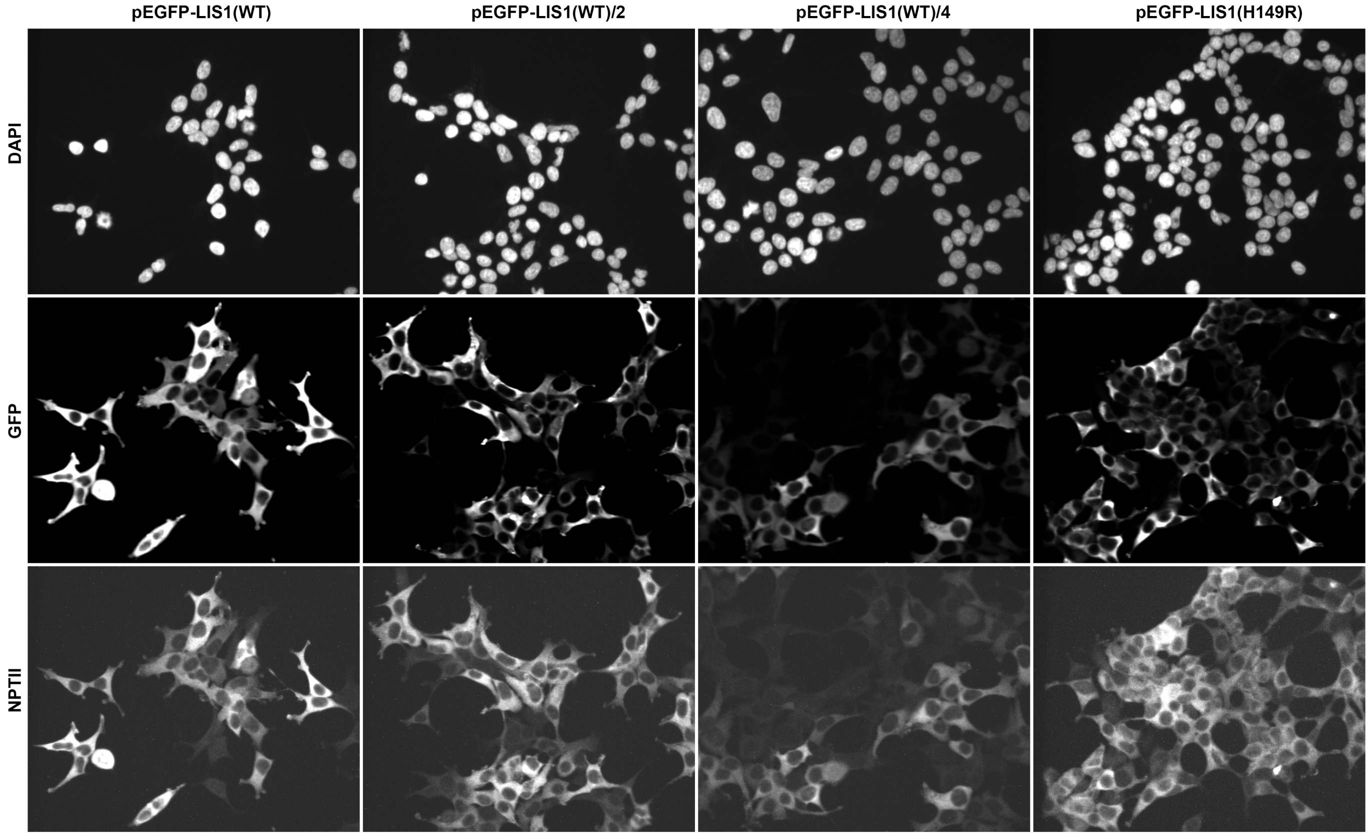

Plasmid	DNA, μg	Transfection efficiency
pEGFP-LIS1(WT)	4	91.67±4.06%
pEGFP-LIS1(WT)	2	89.61±3.41%
pEGFP-LIS1(WT)	1	87.72±8.08%
pEGFP-LIS1(H149R)	4	87.14±7.60%

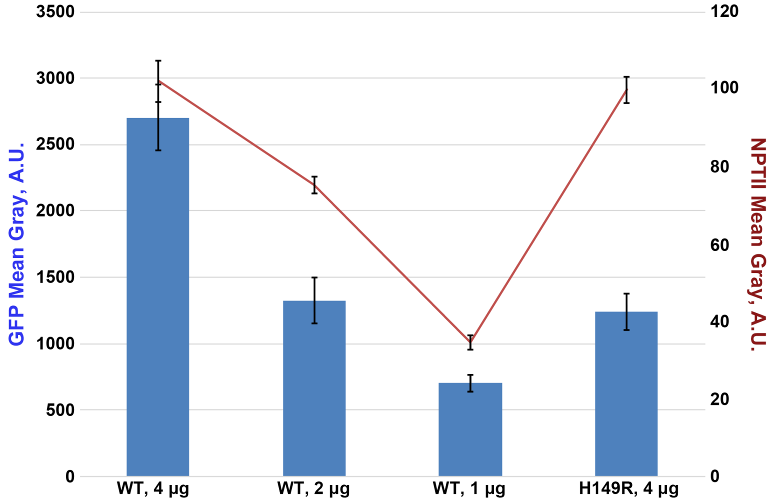

Figure S3: LIS1(H149R) is expressed at lower level compared to wild type LIS1. HEK293 cells were transfected with 1, 2 or 4 μg of pEGFP-LIS1(WT) or 4 μg of pEGFP-LIS1(H149R). 48 hr. after transfection cells were fixed with 3.7% PFA and stained with DAPI, anti-GFP, and anti-neomycin phosphotransferase II (NPTII) antibodies. NPTII is transcribed from a Neomycin resistance gene and its expression in the pEGFP plasmid is driven by an SV40 promoter, independent of EGFP fusion proteins driven by a CMV promoter. Stepwise reduction in the amount of pEGFP-LIS1(WT) plasmid used for transfection results in the graded reduction of both EGFP-LIS1(WT) (blue bars) and NPTII (red line) fluorescence intensity. Similar transfection efficiency* achieved between constructs at all plasmid concentrations indicates that EGFP-fusion protein levels reflect EGFP-protein turnover (translation or stability) as opposed to fewer cells being transfected. For pEGFP-LIS1(WT) there is a proportional correlation between EGFP-LIS1(WT) (blue bars) and NPTII (red line) protein immunofluorescence signals, both of which are reduced as less plasmid is used for transfection. 4 μg pEGFP-LIS1(H149R) has the same NPTII protein signal (red line) as 4 g pEGFP-LIS1(WT). However, EGFP-LIS1(H149R) expression signal matches that of EGFP-LIS1(WT) signal at 2 μg of pEGFP-LIS1(WT), suggesting that the H149R mutation reduces the expression level of LIS1 by 50%.

* The transfection efficiency of this di-genic vector was calculated as 100 x (number of neomycin phosphotransferase II (NPTII)-positive cells / number of DAPI-stained nuclei)

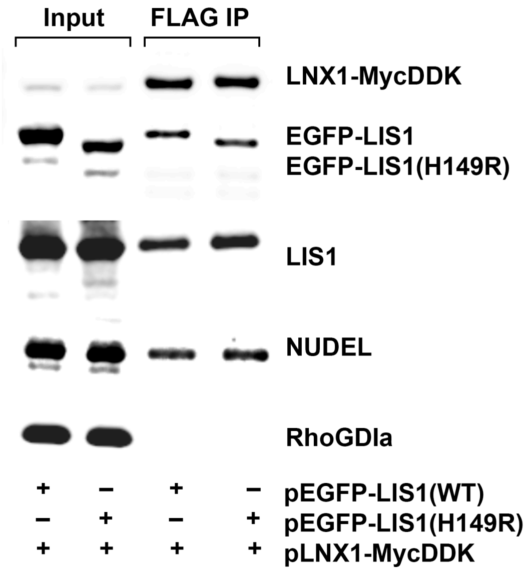

Figure S4: LIS1_H149R mutation associated with human lissencephaly does not affect LIS1-LNX1 interaction. Anti-FLAG pull-down from 293T cells co-transfected with pEGFP-LIS1(WT), pEGFP-LIS1(H149R) and pLNX1-MycDDK, showed similar co-precipitation of EGFP-LIS1(WT) and EGFP-LIS1(H149R) with LNX1-MycDDK and NUDEL (positive control), but not RhoGDI (negative control). Thus, the blot demonstrates the capacity for the missense mutant LIS1 protein to bind LNX1, known LIS1 binding partner NUDEL, and to homodimerize with LIS1 relative to the wild type LIS1. **(**Note: DDK encodes an amino acid sequence identical to FLAG peptide)

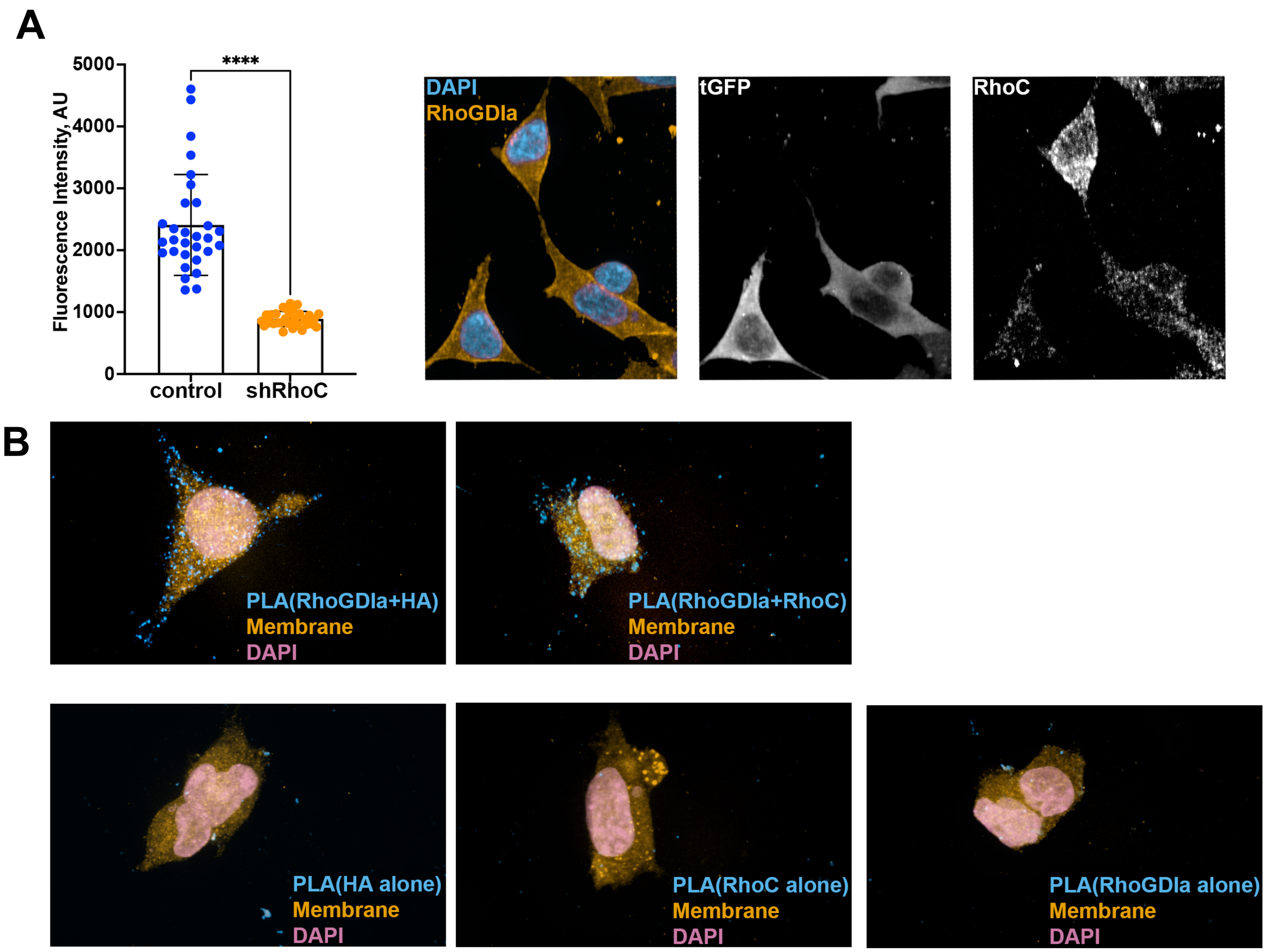

Figure S5: Validation of mouse anti-RhoC antibody for immunofluorescence and specificity of Proximity Ligation Assay. **A.** HEK293T cells were transduced with a tGFP-expressing lentivirus to knock-down RhoC. DAPI (blue) and RhoGDIa (orange) staining is used to identify all cells and tGFP (white) staining is used to identify transduced cells. There is significant reduction in RhoC staining in tGFP-positive cells transduced with shRNAi against *Rhoc* compared to control tGFP-negative untransduced cells. Graph quantifies results from $n = 3$ biologically independent samples, $^{****}P < 0.0001$. Data are presented as the mean ± s.d. **B**. PLA in HEK293T cells using full complement of probes HA(Cdc42)/RhoGDIa or RhoC/RhoGDIa or just one of the two HA(Cdc42), RhoC or RhoGDIa probes. Omitting one of the two probes results in nearly complete loss of the PLA signal, indicating specificity of the signal when both probes are present. DAPI (purple), PLA signal (blue), plasma membrane (orange).

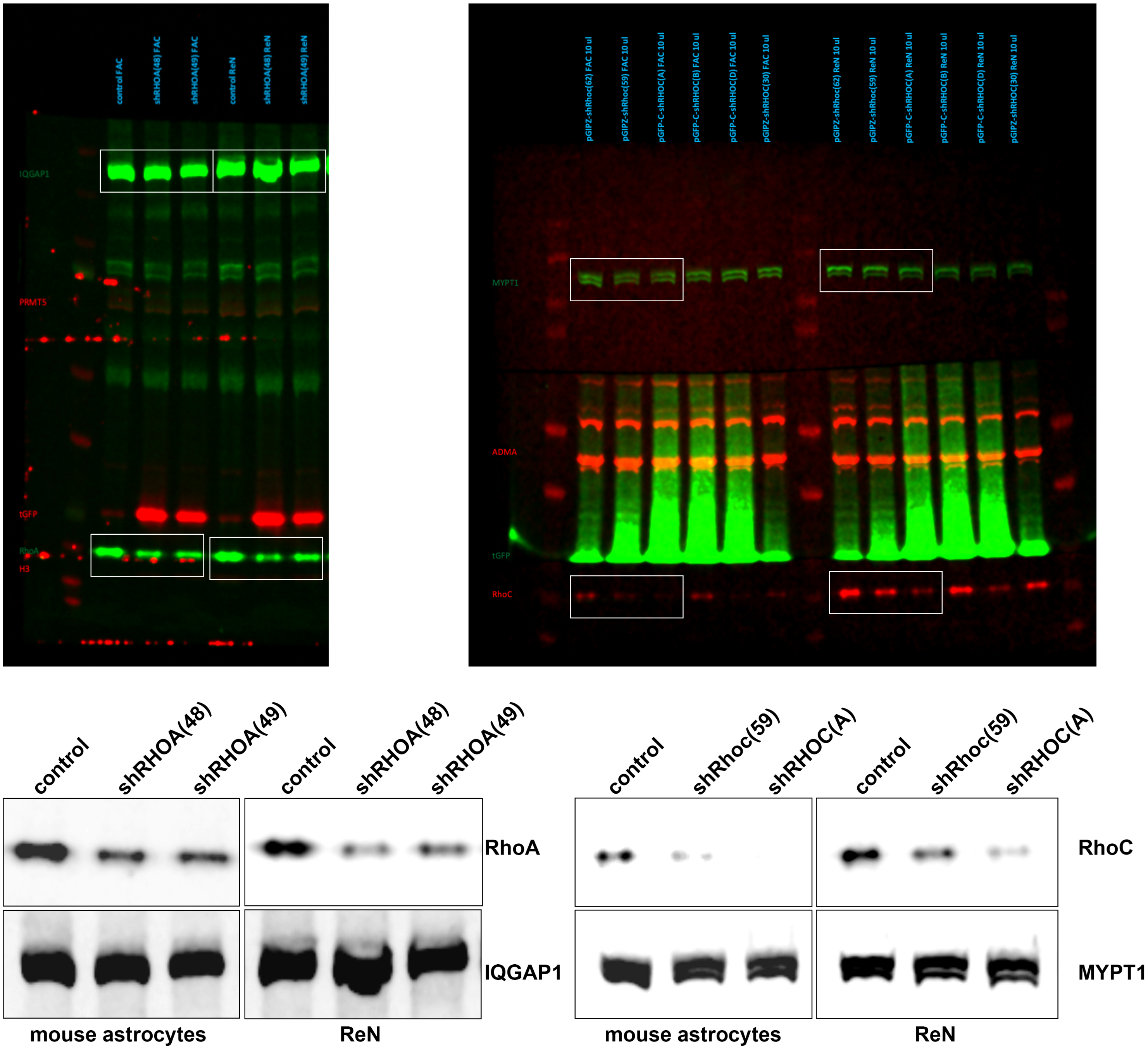

Figure S6: Validation of shRNAi constructs against human RHOA and RHOC and mouse Rhoa and Rhoc mRNA. Primary dissociated forebrain astrocytes and human neuropethelium ReN cells were transduced with control, pGIPZ-shRHOA(48) (Horizon Discovery, RHS4430-200223832), pGIPZ-shRHOA(49) (Horizon Discovery RHS4430-200226019), pGIPZ-shRHOC(59) (Horizon Discovery, RMM4431-200405470) or pGFP-C-shRHOC(A) (OriGene, TL302002A) lentiviral particles. 3 days after transduction cells were lysed and analyzed by Western Blot. Transduction with anti-RHOA shRNA specifically reduced RhoA, and transduction with anti-Rhoc shRNA specifically reduced RhoC protein. IQGAP1 and MYPT1 were used as loading controls.

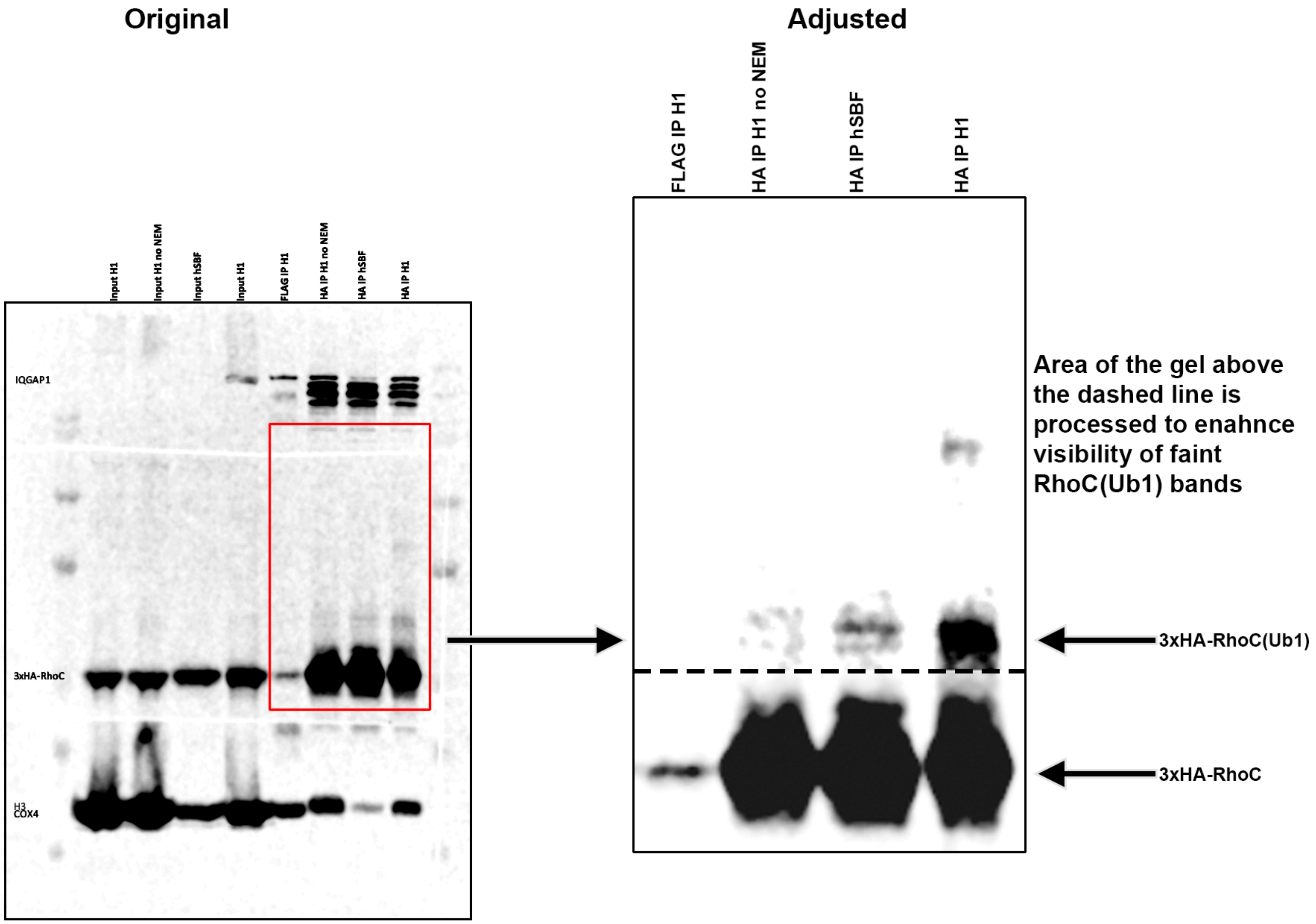

Figure S7: Presence of mono-ubiquitinated RhoC in adult human skin fibroblast (hSBF) and human embryonic stem cells (H1). Cultured hSBF and H1 cells were transduced with pLenti-3xHA-RhoC(WT) lentiviral particles. 5 days after transduction cells were lysed in the absence (negative control) or presence of 20 mM NEM to stabilize ubiquitinated proteins. 3xHA-RhoC was then immunoprecipitated using anti-FLAG (negative control) or anti-HA antibody conjugated magnetic beads. Immunoprecipitated proteins were analyzed by Western Blot. In both hSBF and rapidly dividing H1 cells mono-ubiquitinated RhoC can be detected.

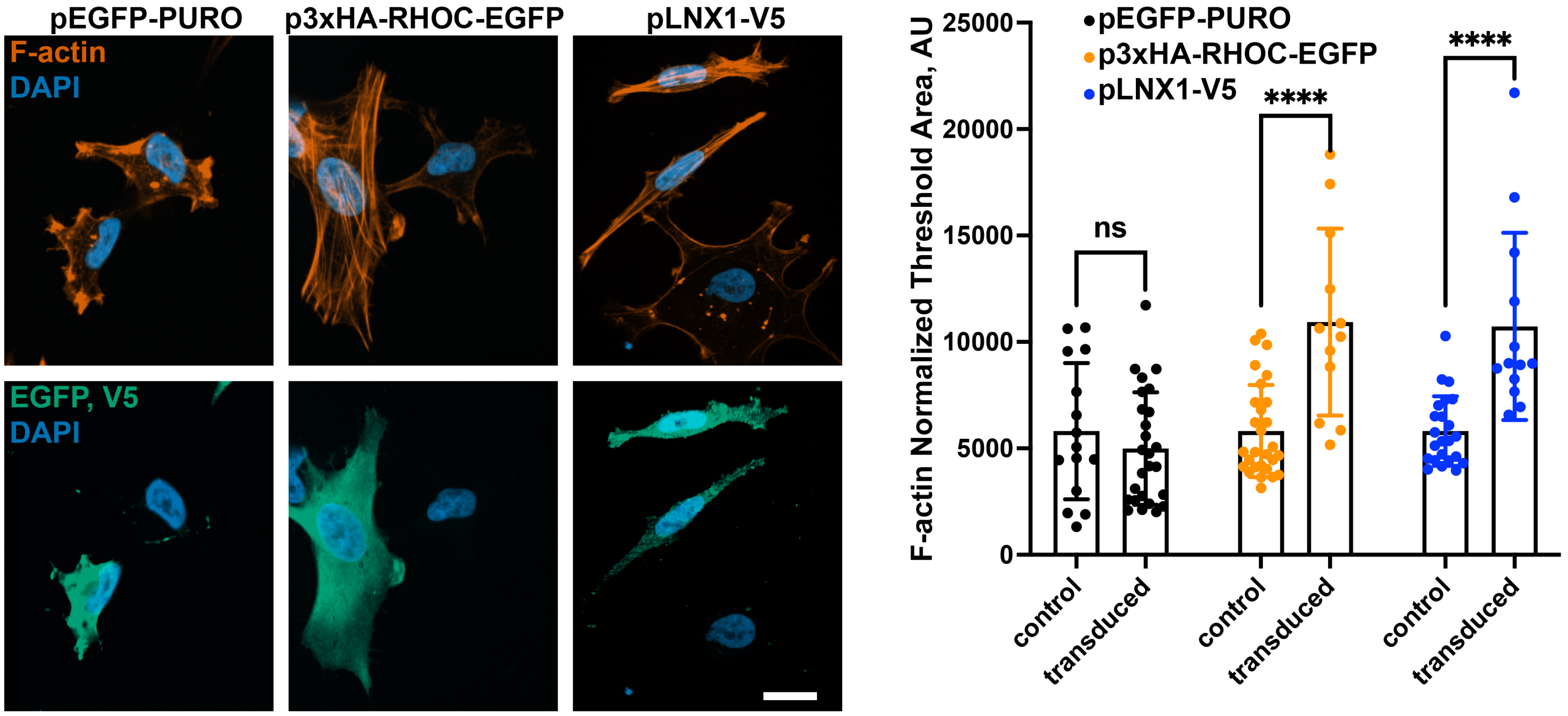

Figure S8: Filamentous actin content (phalloidin, orange) of ReN cells transduced with a lentivirus to overexpress RhoC or LNX1. Immunolabels with anti-GFP or anti-V5 (green) identify cells that are transduced. Scale bar, 10 µm. Graph quantifies results from data pooled over three biologically independent samples: pEGFP-PURO Control, n=15 cells, pEGFP-PURO Transduced, n=26 cells; p3xHA-RHOC-EGFP Control, n=28 cells, p3xHA-RHOC-EGFP Transduced, n=12 cells; pLNX1-V5 Control, n=23 cells, pLNX1-V5 Transduced, n=13 cells; ns=not significant with P=0.5177, ****P < 0.0001. Data are presented as the mean ± s.d. Statistical significances were obtained using two-tailed Mann–Whitney rank sum test.

Fig 1a long exposure

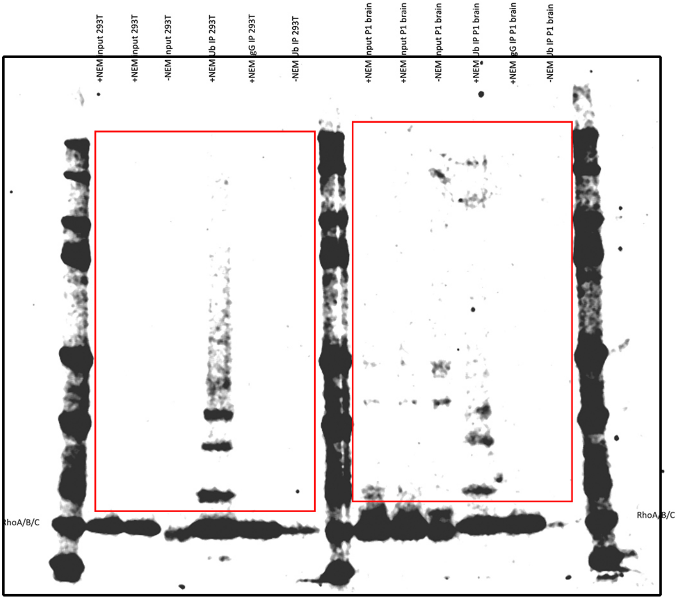

Figure 1a Short exposure H3 and RhoA/B/C bands

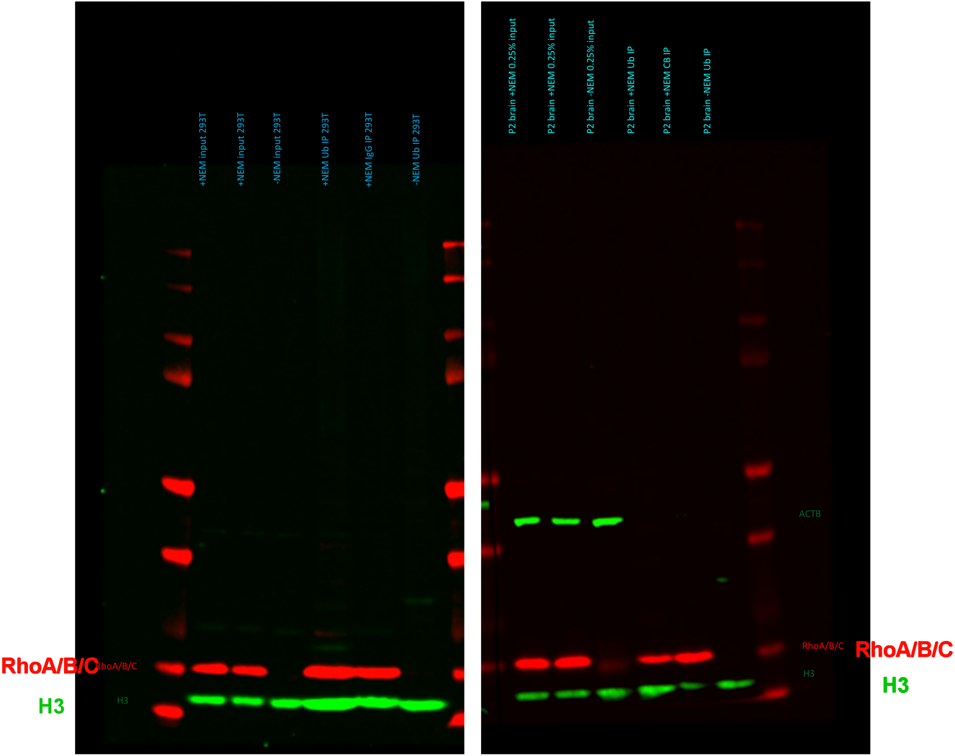

Figure 1a Short exposure

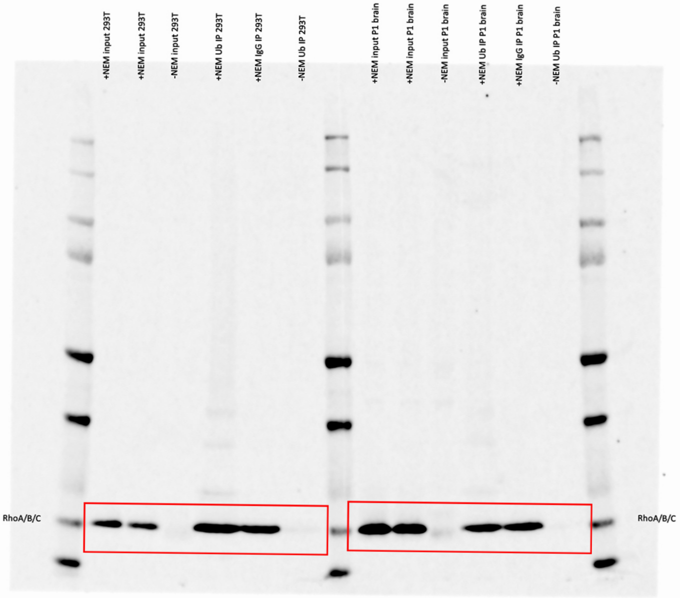

Fig1b pt1

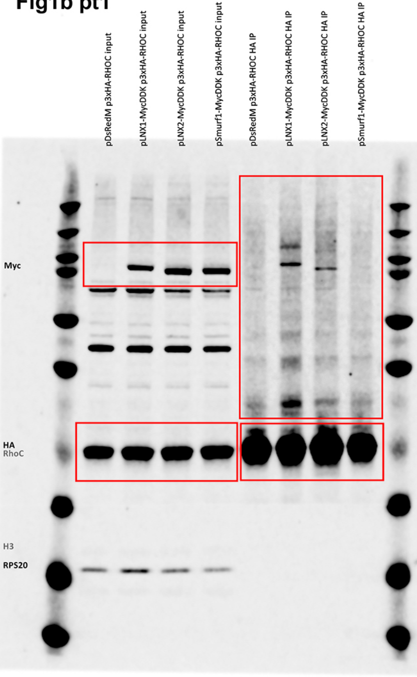
pDsRedM p3xHA-RHOC input
pLNX1-MycDDK p3xHA-RHOC input
pLNX2-MycDDK p3xHA-RHOC input
pSmurf1-MycDDK p3xHA-RHOC input
pDsRedM p3xHA-RHOC HA IP
pLNX1-MycDDK p3xHA-RHOC HA IP
pLNX2-MycDDK p3xHA-RHOC HA IP
pSmurf1-MycDDK p3xHA-RHOC HA IP
Myc
HA
RhoC
H3
RPS20

Fig1b pt.2

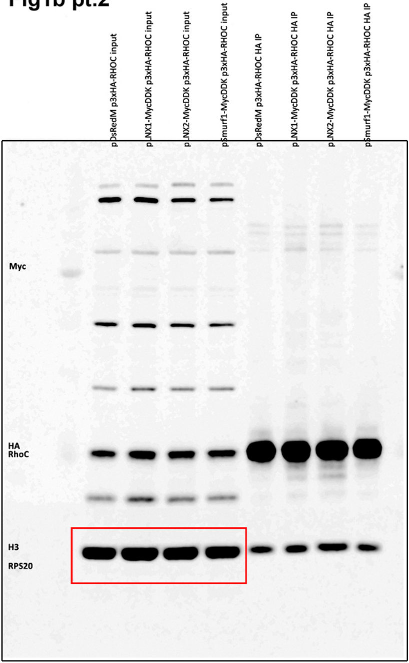

pDsRedM p3xHA-RHOC input
pLNX1-MycDDK p3xHA-RHOC input
pLNX2-MycDDK p3xHA-RHOC input
pSmurf1-MycDDK p3xHA-RHOC input
pDsRedM p3xHA-RHOC HA IP
pLNX1-MycDDK p3xHA-RHOC HA IP
pLNX2-MycDDK p3xHA-RHOC HA IP
pSmurf1-MycDDK p3xHA-RHOC HA IP
Myc
HA
RhoC
H3
RPS20

Fig 1c input

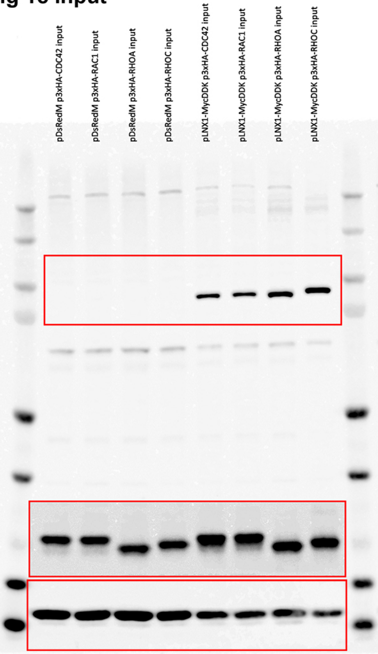
pDsRedM p3xHA-CDC42 input
pDsRedM p3xHA-RAC1 input
pDsRedM p3xHA-RHOA input
pDsRedM p3xHA-RHOC input
pLNX1-MycDDK p3xHA-CDC42 input
pLNX1-MycDDK p3xHA-RAC1 input
pLNX1-MycDDK p3xHA-RHOA input
pLNX1-MycDDK p3xHA-RHOC input

1c input level 1

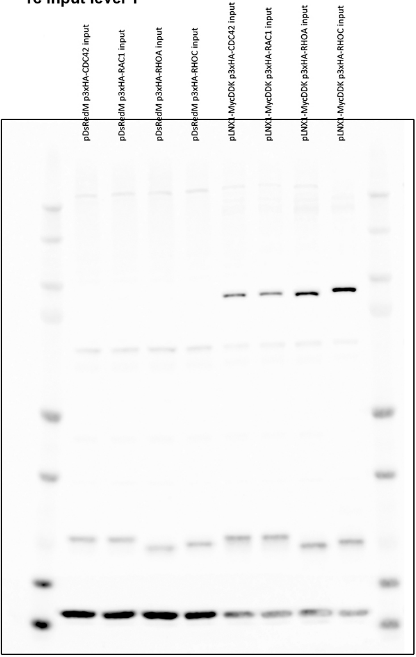

Fig 1c IP

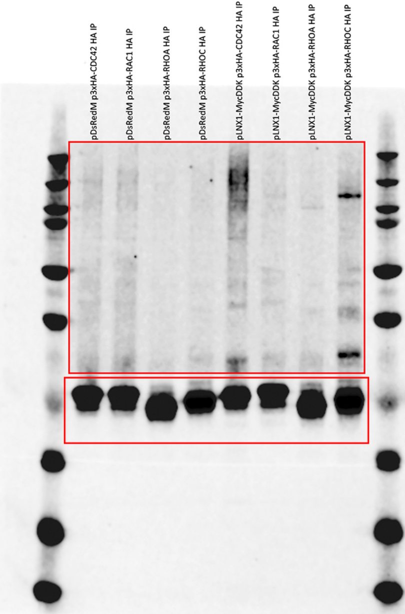
pDsRedM p3xHA-CDC42 HA IP
pDsRedM p3xHA-RAC1 HA IP
pDsRedM p3xHA-RHOA HA IP
pDsRedM p3xHA-RHOC HA IP
pLNX1-MycDDK p3xHA-CDC42 HA IP
pLNX1-MycDDK p3xHA-RAC1 HA IP
pLNX1-MycDDK p3xHA-RHOA HA IP
pLNX1-MycDDK p3xHA-RHOC HA IP

Fig 1d pt.1

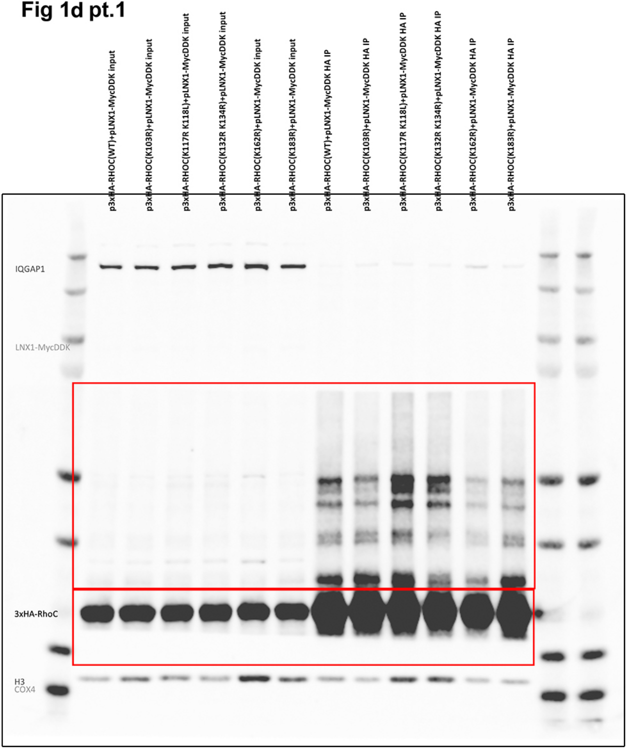
p3xHA-RHOC(WT)+pLNX1-MycDDK input
p3xHA-RHOC(K103R)+pLNX1-MycDDK input
p3xHA-RHOC(K117R K118L)+pLNX1-MycDDK input
p3xHA-RHOC(K132R K134R)+pLNX1-MycDDK input
p3xHA-RHOC(K162R)+pLNX1-MycDDK input
p3xHA-RHOC(K183R)+pLNX1-MycDDK input
p3xHA-RHOC(WT)+pLNX1-MycDDK HA IP
p3xHA-RHOC(K103R)+pLNX1-MycDDK HA IP
p3xHA-RHOC(K117R K118L)+pLNX1-MycDDK HA IP
p3xHA-RHOC(K132R K134R)+pLNX1-MycDDK HA IP
p3xHA-RHOC(K162R)+pLNX1-MycDDK HA IP
p3xHA-RHOC(K183R)+pLNX1-MycDDK HA IP
IQGAP1
LNX1-MycDDK
3xHA-RhoC
H3
COX4

Fig 1d pt.2

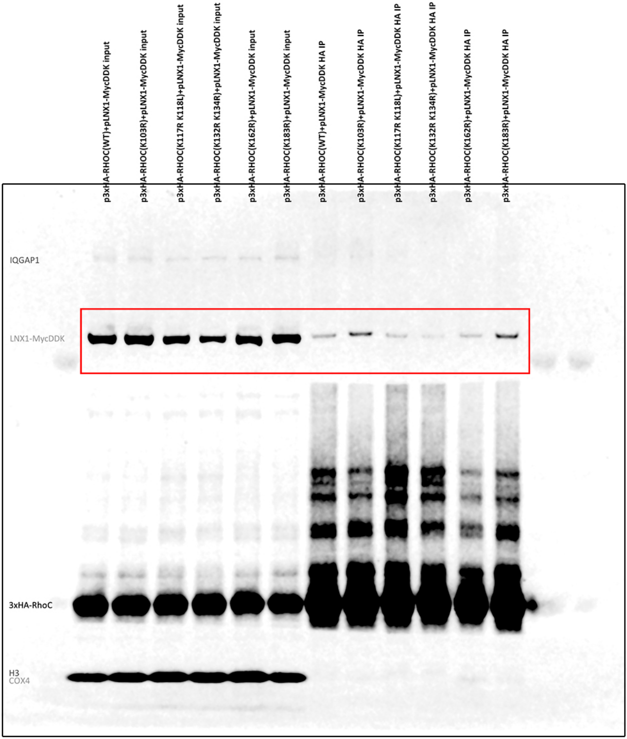
p3xHA-RHOC(WT)+pLNX1-MycDDK input
p3xHA-RHOC(K103R)+pLNX1-MycDDK input
p3xHA-RHOC(K117R K118L)+pLNX1-MycDDK input
p3xHA-RHOC(K132R K134R)+pLNX1-MycDDK input
p3xHA-RHOC(K162R)+pLNX1-MycDDK input
p3xHA-RHOC(K183R)+pLNX1-MycDDK input
p3xHA-RHOC(WT)+pLNX1-MycDDK HA IP
p3xHA-RHOC(K103R)+pLNX1-MycDDK HA IP
p3xHA-RHOC(K117R K118L)+pLNX1-MycDDK HA IP
p3xHA-RHOC(K132R K134R)+pLNX1-MycDDK HA IP
p3xHA-RHOC(K162R)+pLNX1-MycDDK HA IP
p3xHA-RHOC(K183R)+pLNX1-MycDDK HA IP
IQGAP1
LNX1-MycDDK
3xHA-RhoC
H3
COX4

Fig 1e

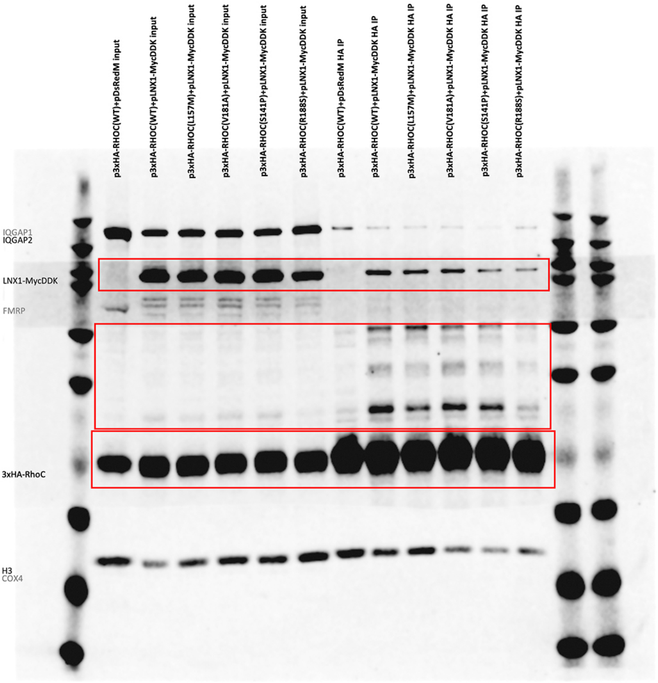
p3xHA-RHOC(WT)+pDsRedM input
p3xHA-RHOC(WT)+pLNX1-MycDDK input
p3xHA-RHOC(L157M)+pLNX1-MycDDK input
p3xHA-RHOC(V181A)+pLNX1-MycDDK input
p3xHA-RHOC(S141P)+pLNX1-MycDDK input
p3xHA-RHOC(R188S)+pLNX1-MycDDK input
p3xHA-RHOC(WT)+pDsRedM HA IP
p3xHA-RHOC(WT)+pLNX1-MycDDK HA IP
p3xHA-RHOC(L157M)+pLNX1-MycDDK HA IP
p3xHA-RHOC(V181A)+pLNX1-MycDDK HA IP
p3xHA-RHOC(S141P)+pLNX1-MycDDK HA IP
p3xHA-RHOC(R188S)+pLNX1-MycDDK HA IP
IQGAP1
IQGAP2
LNX1-MycDDK
FMRP
3xHA-RhoC
H3
COX4

Fig 2a green

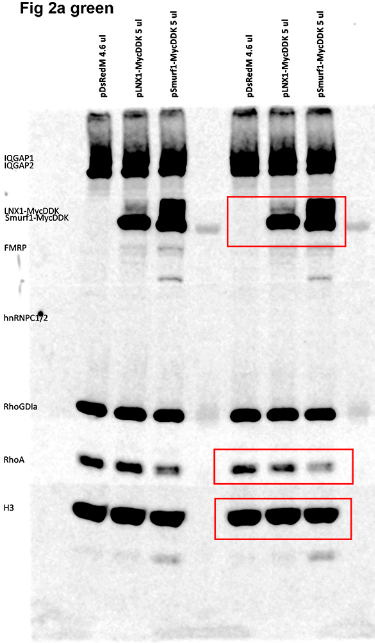
pDsRedM 4.6 ul
pLNX1-MycDDK 5 ul
pSmurf1-MycDDK 5 ul
pDsRedM 4.6 ul
pLNX1-MycDDK 5 ul
pSmurf1-MycDDK 5 ul
IQGAP1
IQGAP2
LNX1-MycDDK
Smurf1-MycDDK
FMRP
hnRNPC1/2
RhoGDIa
RhoA
H3

Fig 2a red

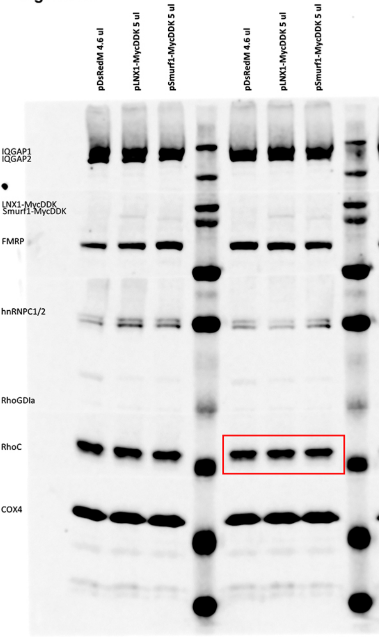

pDsRedM 4.6 ul
pLNX1-MycDDK 5 ul
pSmurf1-MycDDK 5 ul
pDsRedM 4.6 ul
pLNX1-MycDDK 5 ul
pSmurf1-MycDDK 5 ul
IQGAP1
IQGAP2
LNX1-MycDDK
Smurf1-MycDDK
FMRP
hnRNPC1/2
RhoGDIa
RhoC
COX4

Figure 2b RhoA (right side of gel green channel) + GST-Rhotekin

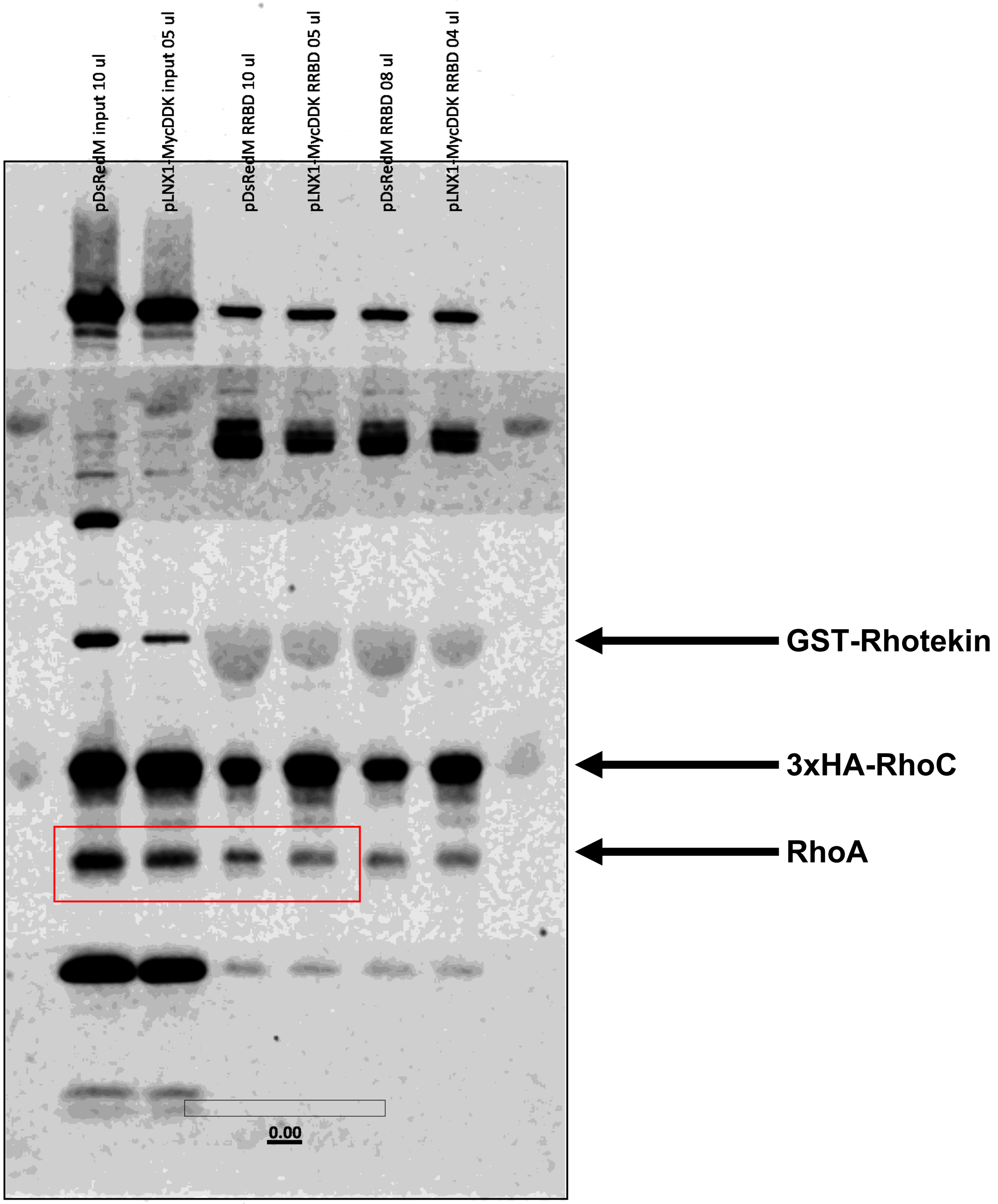

Fig 2b RhoC

(Left side of gel, red channel)

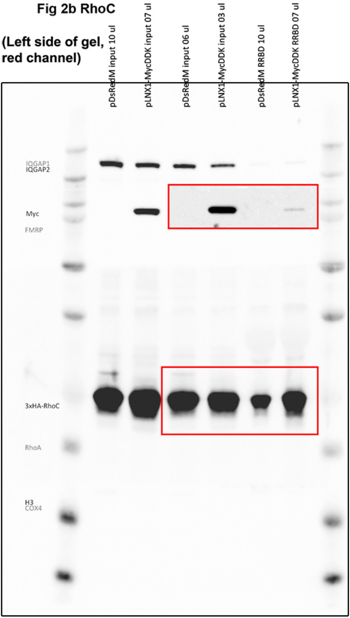

Fig 3a pt 1

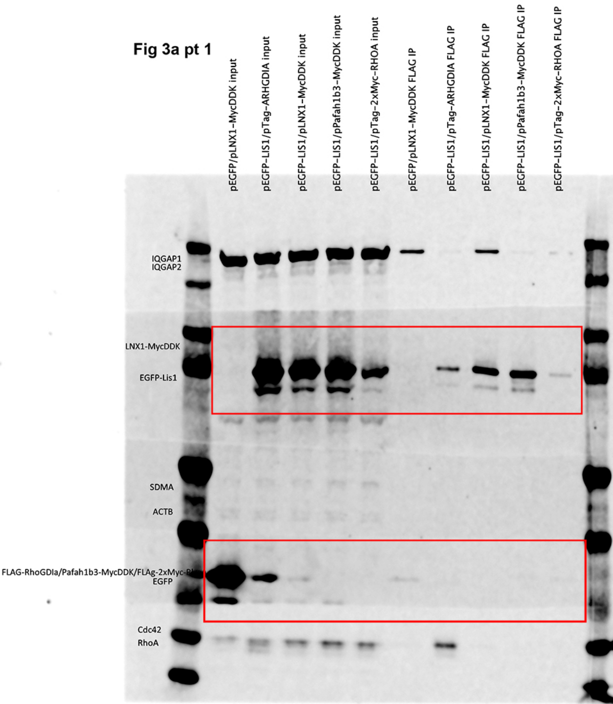
pEGFP/pLNX1-MycDDK input
pEGFP-LIS1/pTag-ARHGDIA input
pEGFP-LIS1/pLNX1-MycDDK input
pEGFP-LIS1/pPafah1b3-MycDDK input
pEGFP-LIS1/pTag-2xMyc-RHOA input
pEGFP/pLNX1-MycDDK FLAG IP
pEGFP-LIS1/pTag-ARHGDIA FLAG IP
pEGFP-LIS1/pLNX1-MycDDK FLAG IP
pEGFP-LIS1/pPafah1b3-MycDDK FLAG IP
pEGFP-LIS1/pTag-2xMyc-RHOA FLAG IP
IQGAP1
IQGAP2
LNX1-MycDDK
EGFP-Lis1
SDMA
ACTB
FLAG-RhoGDIa/Pafah1b3-MycDDK/FLAg-2xMyc-
EGFP
Cdc42
RhoA

Fig 3a Pt.2

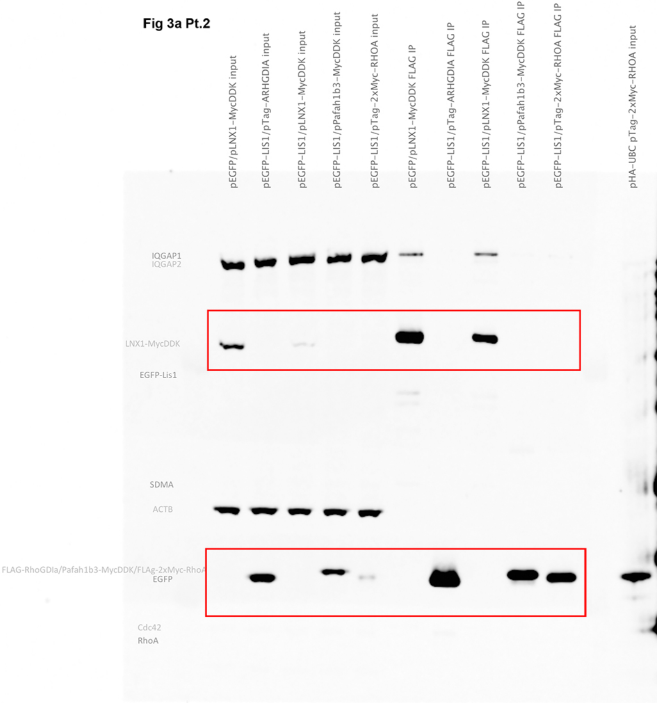
pEGFP/pLNX1-MycDDK input
pEGFP-LIS1/pTag-ARHGDIA input
pEGFP-LIS1/pLNX1-MycDDK input
pEGFP-LIS1/pPafah1b3-MycDDK input
pEGFP-LIS1/pTag-2xMyc-RHOA input
pEGFP/pLNX1-MycDDK FLAG IP
pEGFP-LIS1/pTag-ARHGDIA FLAG IP
pEGFP-LIS1/pLNX1-MycDDK FLAG IP
pEGFP-LIS1/pPafah1b3-MycDDK FLAG IP
pEGFP-LIS1/pTag-2xMyc-RHOA FLAG IP
pHA-UBC pTag-2xMyc-RHOA input
IQGAP1
IQGAP2
LNX1-MycDDK
EGFP-Lis1
SDMA
ACTB
FLAG-RhoGDIa/Pafah1b3-MycDDK/FLAg-2xMyc-RhoA
EGFP
Cdc42
RhoA

Fig 3b 3x-HA-RhoC

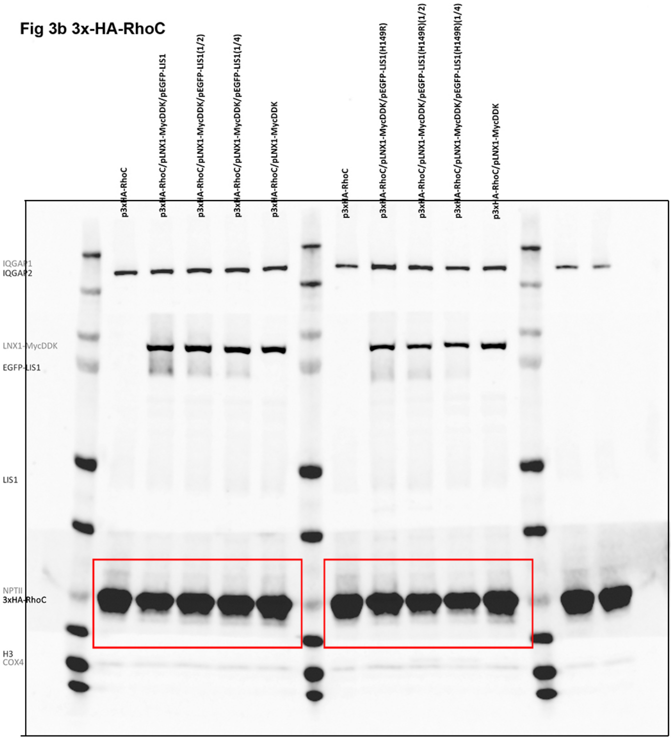

Fig 3b EGFP-Lis1 input

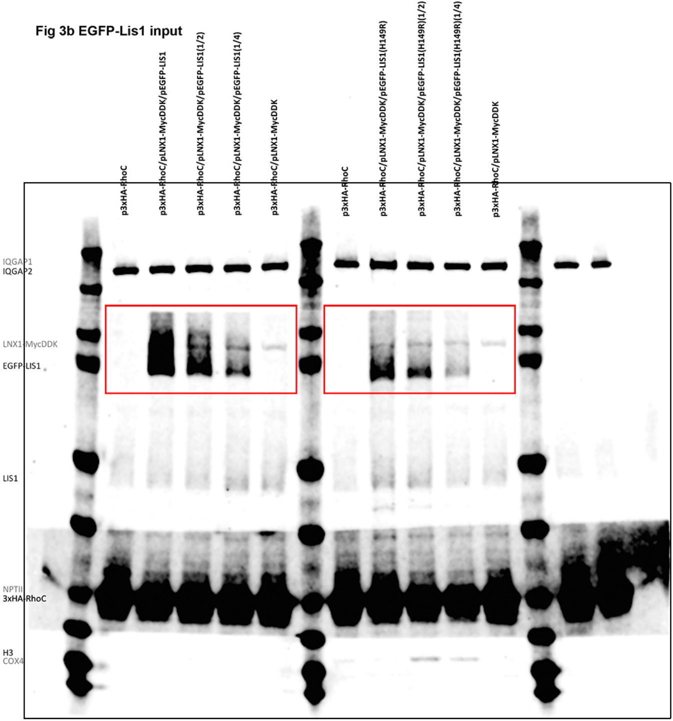

Fig 3b HA IP

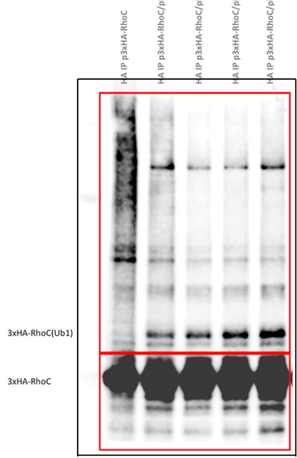

H3
COX4

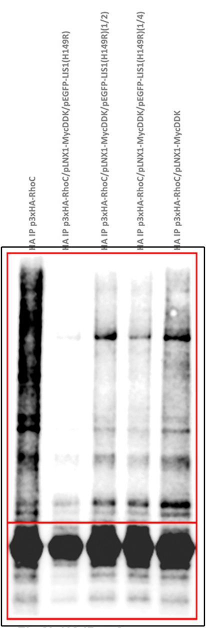

Fig 3b HA IP pt.2

Fig 3b LNX1-MycDDK input

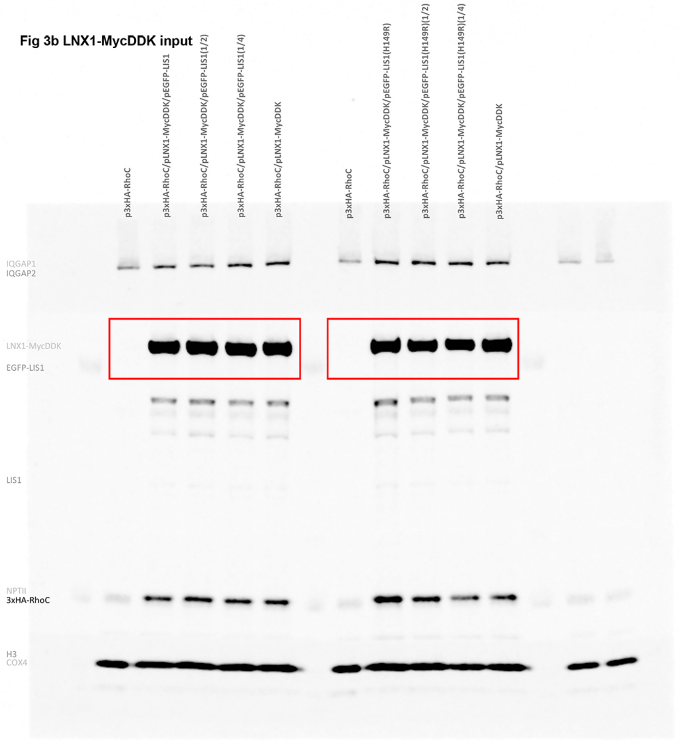

Fig 4a Rho ubiquitination (Green channel)

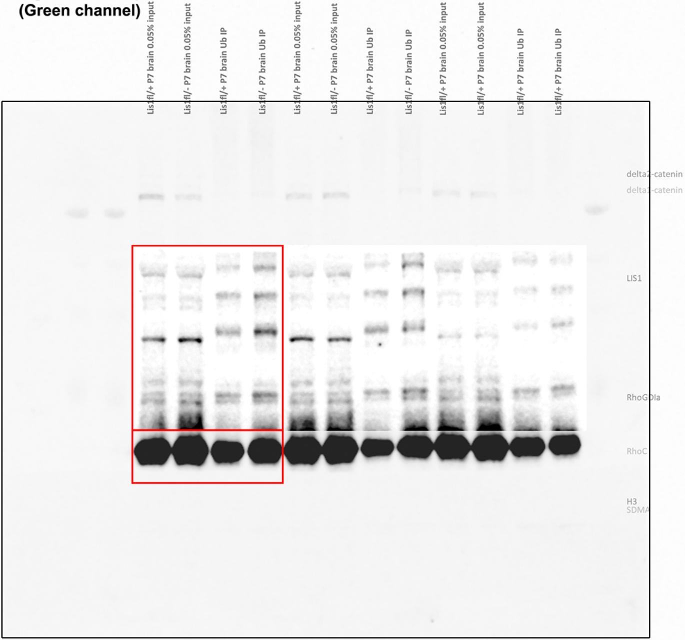

Fig 4a H3
(Red channel)

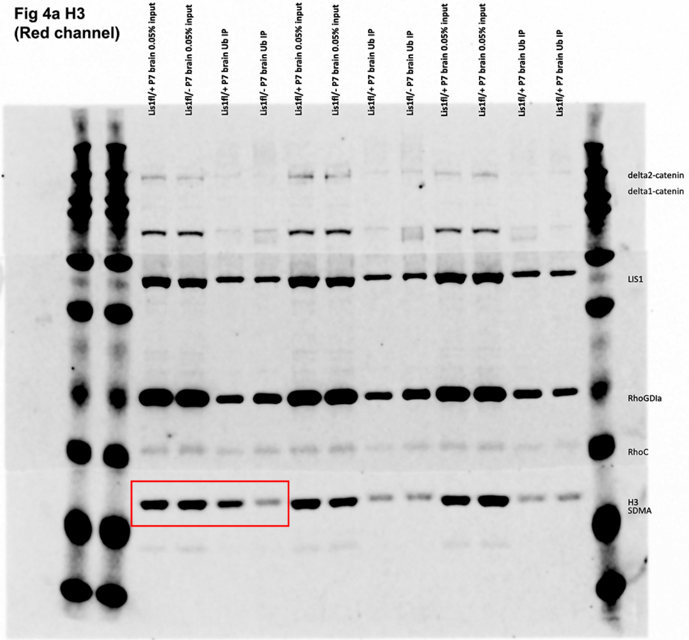

Fig 4a Lis1 (Red channel)

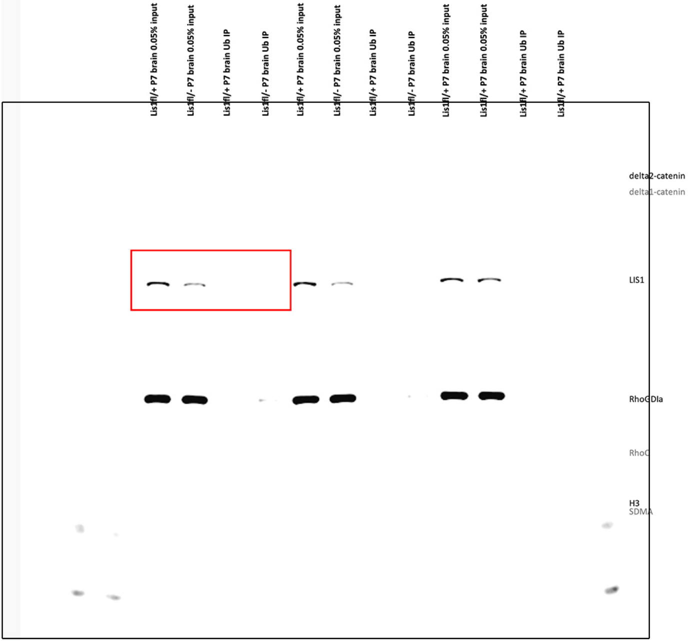

Fig 4b Rac3

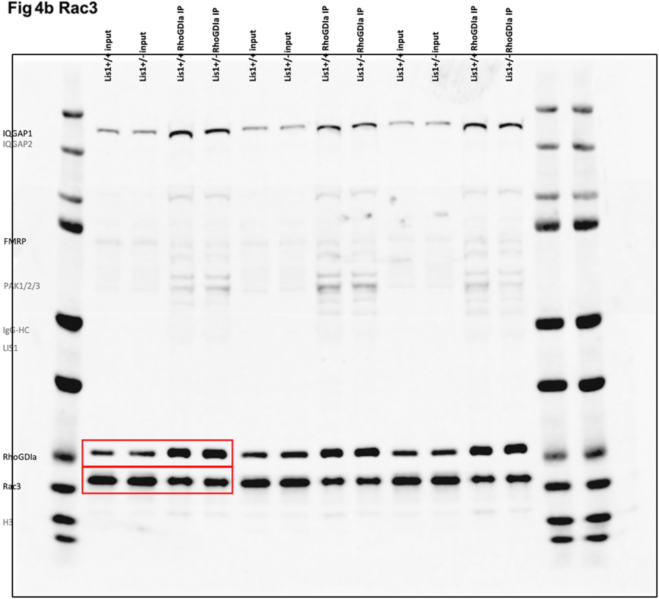
Lis1+/+ input
Lis1+/- input
Lis1+/+ RhoGDIa IP
Lis1+/- RhoGDIa IP
Lis1+/+ input
Lis1+/- input
Lis1+/+ RhoGDIa IP
Lis1+/- RhoGDIa IP
Lis1+/+ input
Lis1+/- input
Lis1+/+ RhoGDIa IP
Lis1+/- RhoGDIa IP
IQGAP1
IQGAP2
FMRP
PAK1/2/3
IgG-HC
LIS1
RhoGDIa
Rac3
H3

Fig 4b Rac3 Lis1 H3

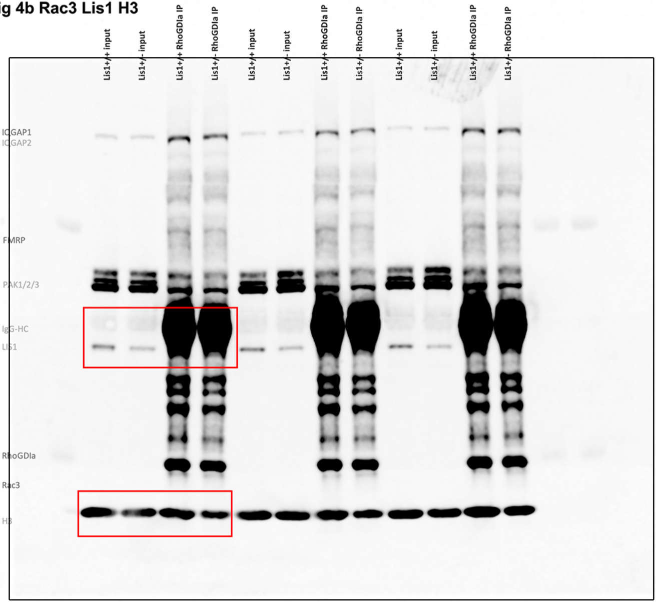

Fig 4b RhoA

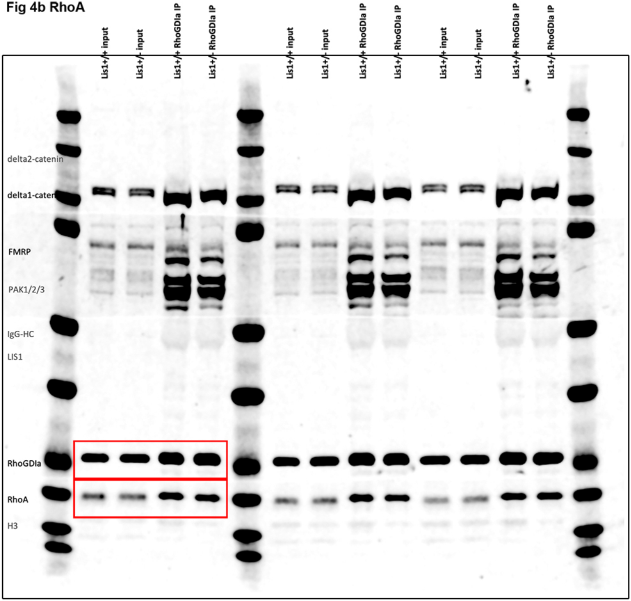

Lis1+/+ input
Lis1+/- input
Lis1+/+ RhoGDIa IP
Lis1+/- RhoGDIa IP
Lis1+/+ input
Lis1+/- input
Lis1+/+ RhoGDIa IP
Lis1+/- RhoGDIa IP
Lis1+/+ input
Lis1+/- input
Lis1+/+ RhoGDIa IP
Lis1+/- RhoGDIa IP
delta2-catenin
delta1-caten
FMRP
PAK1/2/3
IgG-HC
LIS1
RhoGDIa
RhoA
H3

Fig 4b RhoA Lis1 H3

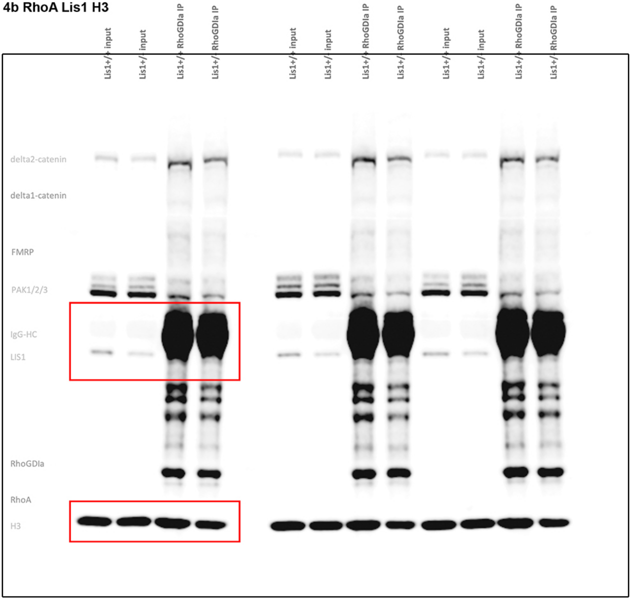

Fig 4b RhoC

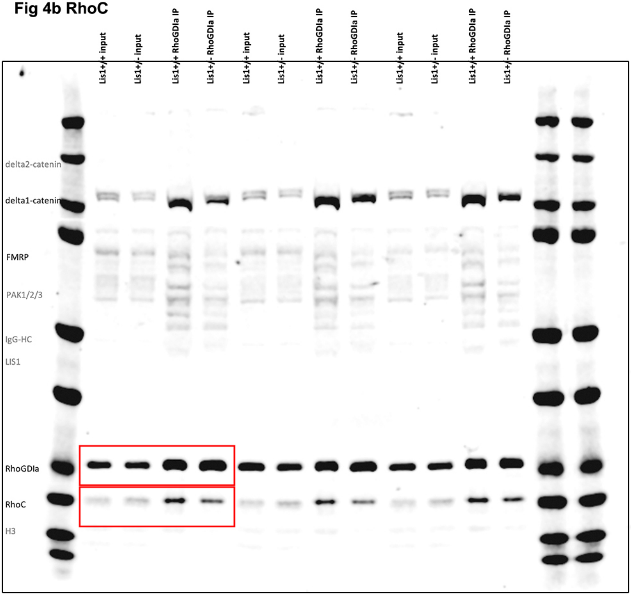
Lis1+/+ input
Lis1+/- input
Lis1+/+ RhoGDIa IP
Lis1+/- RhoGDIa IP
Lis1+/+ input
Lis1+/- input
Lis1+/+ RhoGDIa IP
Lis1+/- RhoGDIa IP
Lis1+/+ input
Lis1+/- input
Lis1+/+ RhoGDIa IP
Lis1+/- RhoGDIa IP
delta2-catenin
delta1-catenin
FMRP
PAK1/2/3
IgG-HC
LIS1
RhoGDIa
RhoC
H3

Fig 4b RhoC Lis1 H3

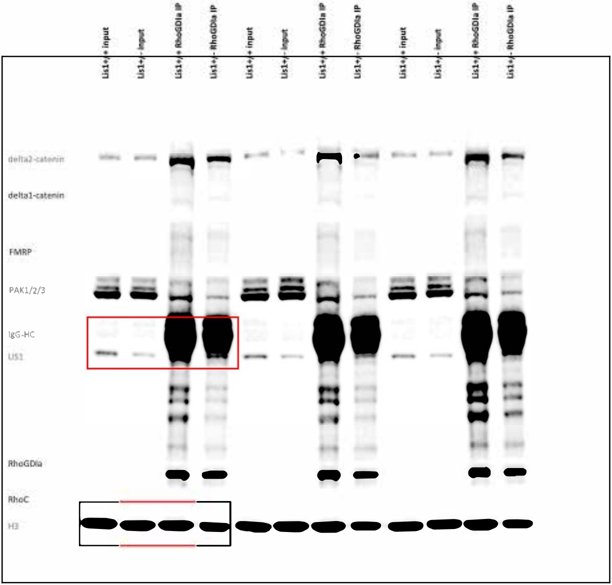

Figure S2 original blots

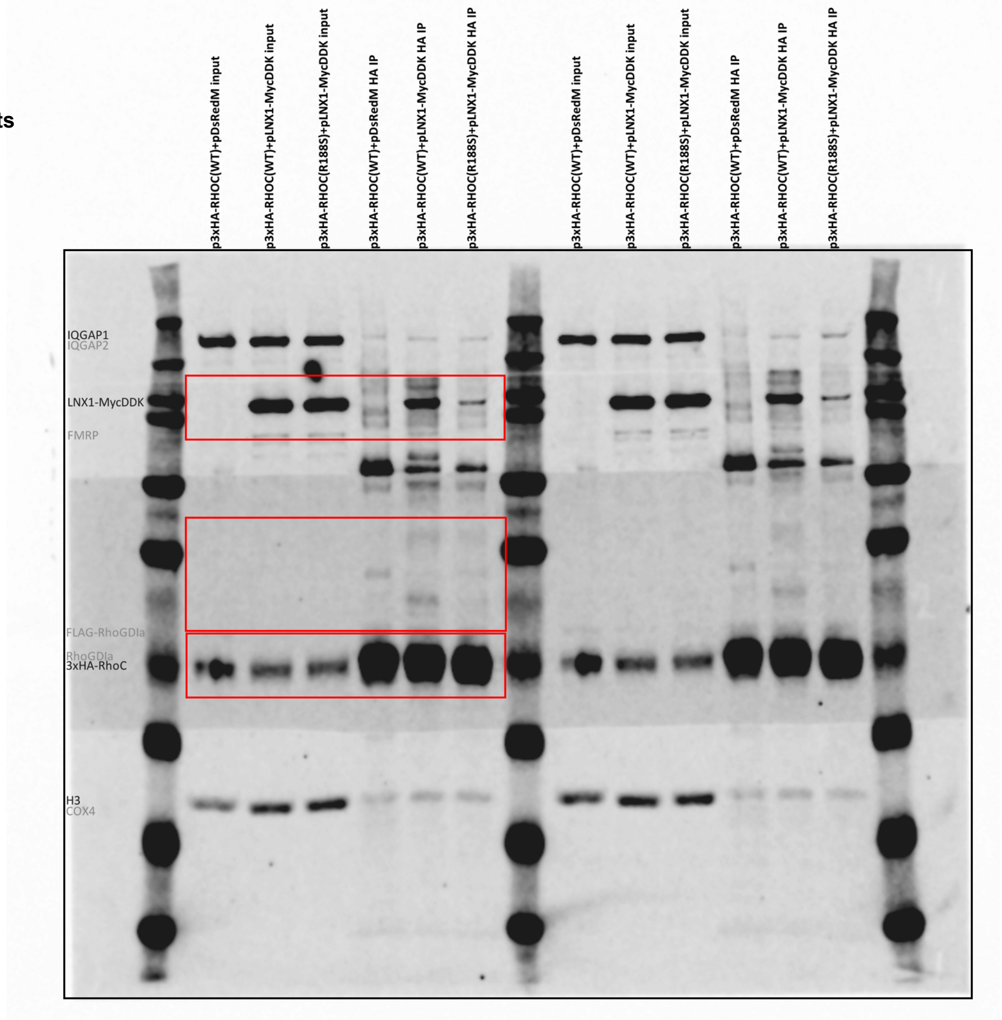

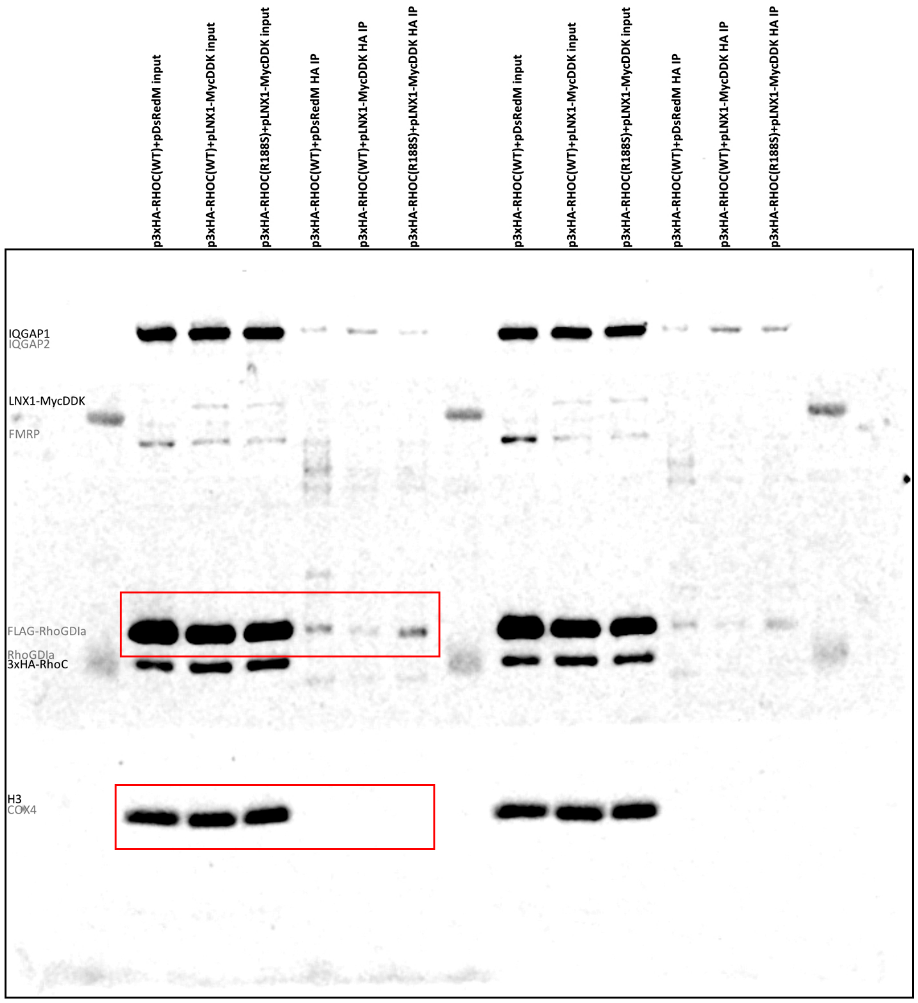

Figure S4 original blots

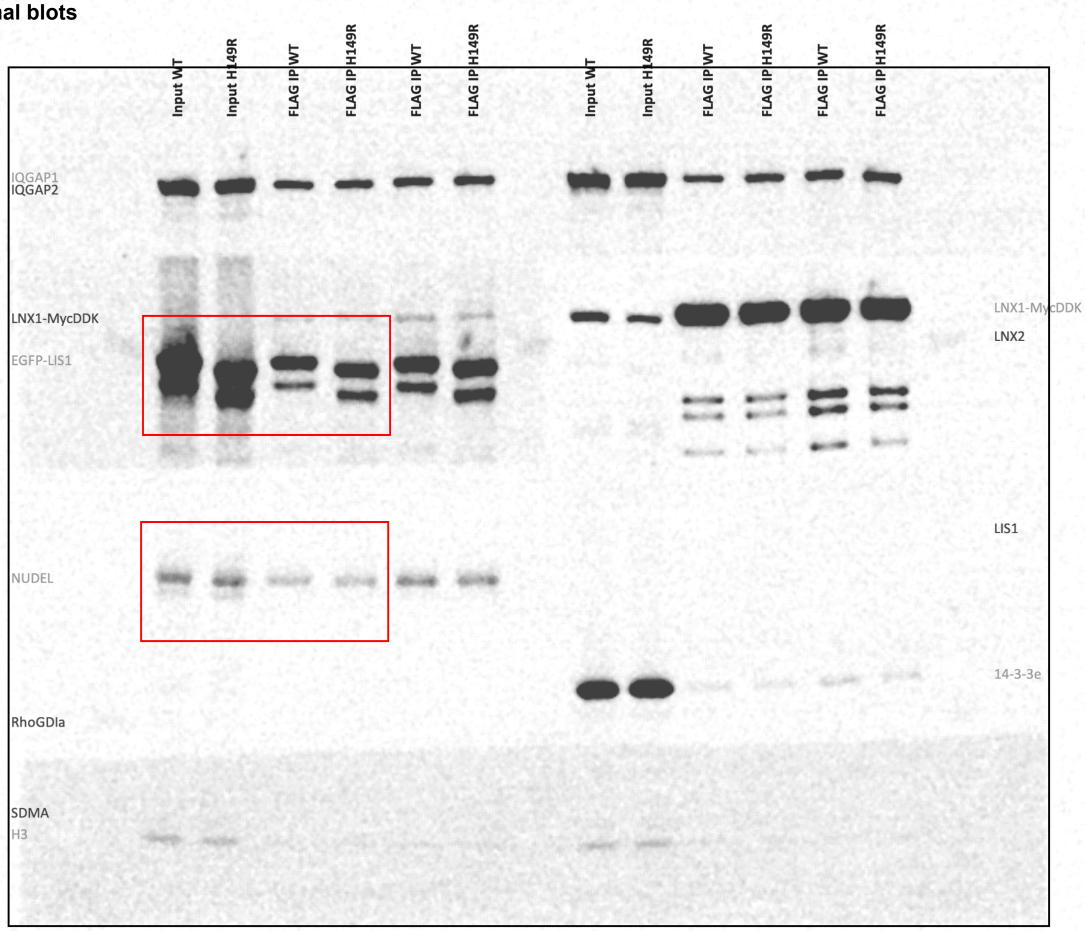

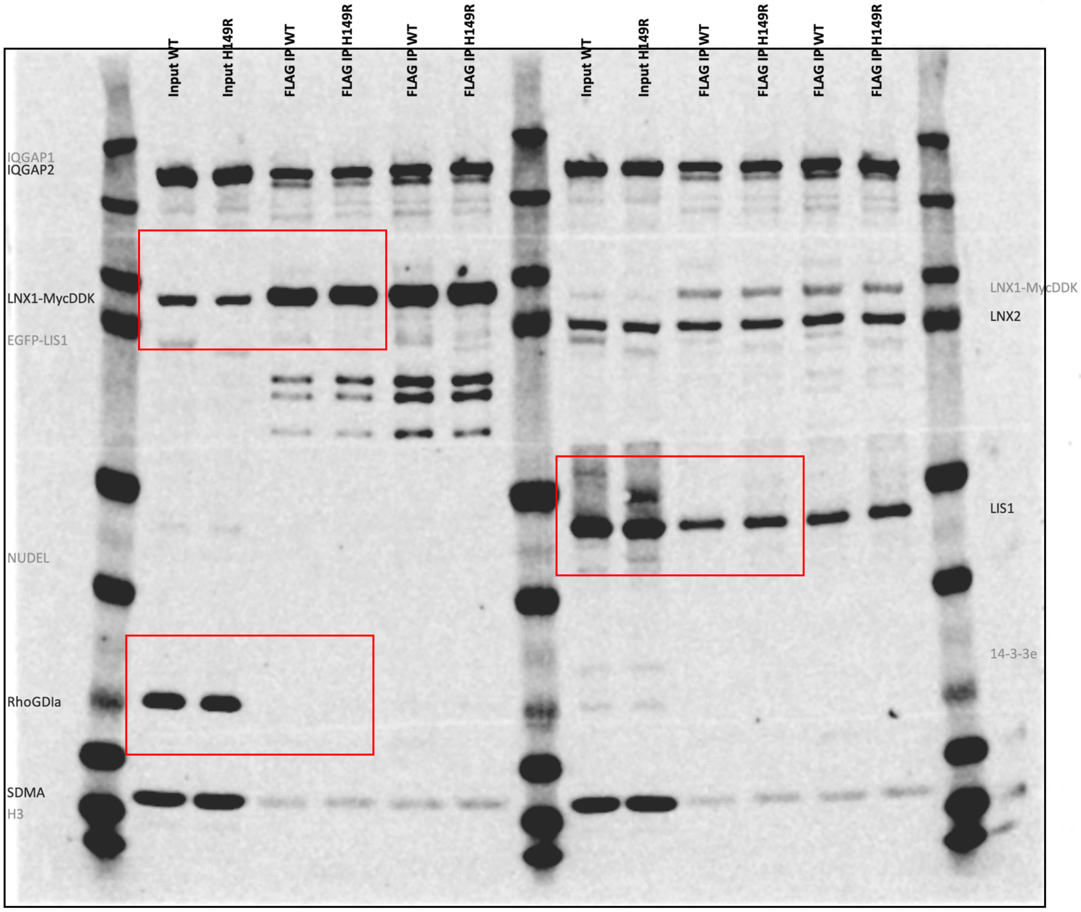

Supplement: Supplementary file 1 — Supplementary Figures. [file 41598_2022_19740_MOESM1_ESM.pdf]
